# Discovery of 2-phenoxyacetamides as inhibitors of the Wnt-depalmitoleating enzyme NOTUM from an X-ray fragment screen†
†This work was first presented at the following international conferences: E. Y. J. Biochemical Society Meeting. Acylation of intracellular and secreted proteins: mechanisms and functional outcomes; 10–12th September 2018; Brighton, UK. P. V. F. EFMC: XXVth International Symposium on Medicinal Chemistry; 2–6th September 2018; Ljubljana, Slovenia.
‡
‡Electronic supplementary information (ESI) available: Synthetic procedures and characterization data; enzyme production and purification methods; OPTS assay procedure; FP-biotin competition assay; TCF/LEF reporter assay; ADME screens; protein crystallization, data collection and structure determination. See DOI: 10.1039/c9md00096h
§
§Atomic coordinates have been deposited in the Protein Data Bank (PDB) and will be released upon publication. PDB ID codes: **3**: 6R8P; **39**: 6R8Q; **45**: 6R8R.


**DOI:** 10.1039/c9md00096h

**Published:** 2019-04-29

**Authors:** Benjamin N. Atkinson, David Steadman, Yuguang Zhao, James Sipthorp, Luca Vecchia, Reinis R. Ruza, Fiona Jeganathan, Georgie Lines, Sarah Frew, Amy Monaghan, Svend Kjær, Magda Bictash, E. Yvonne Jones, Paul V. Fish

**Affiliations:** a Alzheimer's Research UK UCL Drug Discovery Institute , University College London , Cruciform Building, Gower Street , London , WC1E 6BT , UK . Email: p.fish@ucl.ac.uk ; Tel: +44 (0)20 7679 6971; b Division of Structural Biology , Wellcome Centre for Human Genetics , University of Oxford , The Henry Wellcome Building for Genomic Medicine, Roosevelt Drive , Oxford , OX3 7BN , UK . Email: yvonne@strubi.ox.ac.uk ; Tel: +44 (0)1865 287 546; c The Francis Crick Institute , 1 Midland Road , London , NW1 1AT , UK

## Abstract

NOTUM is a carboxylesterase that has been shown to act by mediating the *O*-depalmitoleoylation of Wnt proteins resulting in suppression of Wnt signaling. Here, we describe the development of NOTUM inhibitors that restore Wnt signaling for use in *in vitro* disease models where NOTUM over activity is an underlying cause. A crystallographic fragment screen with NOTUM identified 2-phenoxyacetamide **3** as binding in the palmitoleate pocket with modest inhibition activity (IC_50_ 33 μM). Optimization of hit **3** by SAR studies guided by SBDD identified indazole **38** (IC_50_ 0.032 μM) and isoquinoline **45** (IC_50_ 0.085 μM) as potent inhibitors of NOTUM. The binding of **45** to NOTUM was rationalized through an X-ray co-crystal structure determination which showed a flipped binding orientation compared to **3**. However, it was not possible to combine NOTUM inhibition activity with metabolic stability as the majority of the compounds tested were rapidly metabolized in an NADPH-independent manner.

## Abstract 2

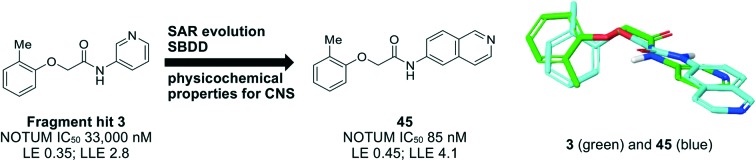
Optimization of fragment hit **3** identified isoquinoline **45** as a potent inhibitor of NOTUM with an unexpected flipped binding mode.

## 

Members of the Wnt family are secreted signaling proteins that play key roles in adult stem cell biology as well as in embryonic development.^1^ Wnts initiate signaling by binding to cell surface receptors and two main pathways have been identified downstream of Wnts: the so-called ‘canonical’ and ‘planar cell polarity’ pathways. These pathways are triggered by the binding of Wnt to a member of the Frizzled family of cell surface receptors and, for the canonical pathway, a member of the LDL-receptor-related protein (LRP) family of cell surface receptors (typically LRP5 or LRP6). This binding elicits an intracellular signaling cascade that results in both biochemical and transcriptional changes within the cell, with the canonical pathway involving the accumulation and translocation of β-catenin. Both pathways are tightly regulated by a sophisticated network of modulators and feedbacks including secreted inhibitory Dickkopf (DKK) proteins^2^ and post translational modifications (PTM).^3^,^4^

Conversely, dysregulation of Wnt signaling is frequently associated with growth-related pathologies and cancers,^5^ particularly those of tissues for which Wnts normally stimulate self-renewal and repair. Wnt signaling is also implicated to have a role in neurodegenerative diseases such as Alzheimer's disease (AD). Cognitive impairments, characteristic of AD, correlate closely with the loss of synapses and current knowledge suggests that excess amyloid-β (Aβ) causes synapse dysfunction by impairing synapse maintenance, at least in part, through causing dysfunction of Wnt signaling.^6^,^7^ Compromised Wnt signaling may also be associated with AD through loss of blood–brain barrier (BBB) integrity^8^ and Aβ generation through β-secretase (BACE1) expression.^9^

*O*-Palmitoleoylation of a conserved serine residue in Wnt proteins is a key PTM required for efficient binding of Wnt proteins to Frizzled receptors, a requirement for signal transduction.^10^ A carboxylesterase NOTUM was previously shown to antagonize Wnt signaling pathways.^11^ More recently, NOTUM has been shown to act by mediating the depalmitoleoylation of Wnt proteins resulting in suppression of Wnt signaling.^12^,^13^ It follows that inhibition of NOTUM could restore Wnt signaling with potential benefit in disease where Wnt deficiency is an underlying cause.

There are only a few reports of NOTUM inhibitors with compounds falling into two main categories (Scheme 1). Thienopyrimidine **LP-922056** (**1**) is a potent, orally active inhibitor of NOTUM that has shown NOTUM to be a potential drug target for stimulating bone formation and treating osteoporosis.^14^ However, although **1** demonstrates low clearance and good bioavailability, the structure contains an essential carboxylic acid and acids tend to have low passive brain penetration;^15^–^18^ in fact, it is an accepted strategy to add a carboxylic acid group to minimize brain penetration of drugs for peripheral targets.^19^*N*-Hydroxyhydantoin carbamates are potent and selective irreversible inhibitors of NOTUM discovered by activity-based protein profiling (ABPP).^20^ An optimized inhibitor **ABC99** (**2**) preserves Wnt-mediated cell signaling in the presence of NOTUM and was converted to a clickable ABPP probe for visualizing NOTUM in biological systems. Due to their reactive mechanism of action forming covalent adducts with NOTUM Ser232, it is unlikely that these compounds will be suitable for *in vivo* studies. Hence, our objective was to discover potent small molecule inhibitors of NOTUM suitable for exploring the regulation of Wnt signaling in the central nervous system (CNS) and modulation of AD phenotypes.

**Scheme 1:**
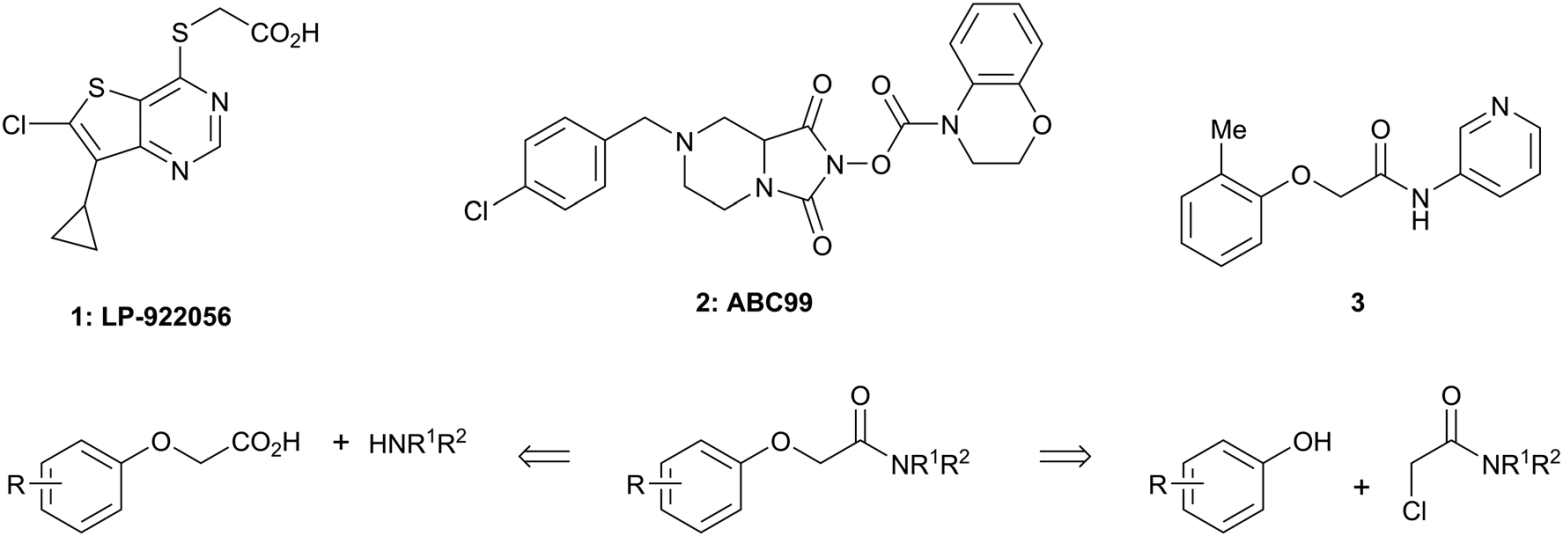
Chemical structures of LP-922056 (**1**), ABC99 (**2**) and initial fragment hit **3**. General scheme for the synthesis of 2-phenoxyacetamides reported in Tables 1 and 2.

In order to identify new small molecule inhibitors of NOTUM, a crystallographic fragment screen was performed using the XChem platform at Diamond Light Source. Crystals of C-terminal his-tagged NOTUM(Ser81-Thr451 Cys330Ser) were soaked with the DSI-Poised library (XChem, 768 fragments).^21^ Crystal structures of NOTUM show a distinctive pocket that accommodates the palmitoleate group (Fig. 1A).^12^ Fragments observed to bind in the palmitoleate pocket were all re-synthesized as solid samples to establish structure and purity. Inhibition of NOTUM carboxylesterase activity of these hits was measured in a cell-free biochemical assay. In brief, test compounds (dispensed to give 10 point concentration–response-curves) were incubated with NOTUM(81-451 Cys330Ser) and trisodium 8-octanoyloxypyrene-1,3,6-trisulfonate (OPTS) as the substrate for 1 h, and fluorescence recorded; an inhibitor of NOTUM would suppress fluorescence by binding to NOTUM and preventing hydrolysis of OPTS (ESI,‡ Fig. S1).^14^,^22^

**Fig. 1:**
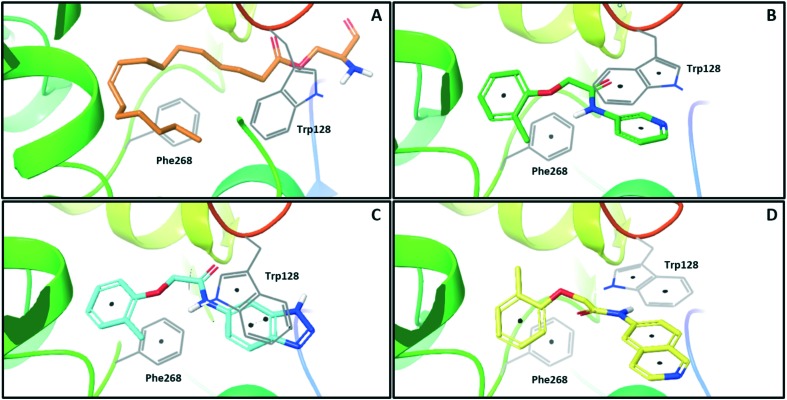
X-ray crystal structures of ligands bound to NOTUM. (A) *O*-Palmitoleoyl serine (orange) (S232A mutant NOTUM), PDB ID: ; 4UZQ; (B) fragment hit **3** (green) with key pi–pi stacking interactions between the ligand and Trp128 and Phe268 indicated, PDB ID: ; 6R8P; (C) benzotriazole **39** (blue) showing a similar binding mode to **3**, but with flipping of Trp128, PDB ID: ; 6R8Q; (D) isoquinoline **45** (yellow) demonstrates a reorientation of the phenoxy and amide groups but with key pi–pi stacking interactions maintained, PDB ID: ; 6R8R. Ligands are shown as sticks, and protein represented as ribbons with key amino acids shown as lines. Key pi–pi stacking interactions are shown between the ligands and the protein as black dots. Phe320 lies above the plane of the figure and is not shown for clarity.

One of the preferred hits from this set was 2-(2-methylphenoxy)-*N*-(pyridine-3-yl)acetamide (**3**) (IC_50_ 33 ± 4.7 μM) which was selected for further investigation for several reasons: (1) excellent “hit-like” properties (mw 242; clog *P* 1.7; TPSA 51; LE = 0.35; LLE = 2.8) with no obvious reactive groups; (2) chemically enabled to selectively functionalize each position to explore structure activity relationships (SAR); (3) successful co-crystal structure with NOTUM to support a structure based drug design (SBDD) program; and (4) structural features and physicochemical properties consistent with CNS drug-like space^23^ including a favorable ‘CNS multiparameter optimization’ (CNS MPO) score (CNS MPO = 5.6/6.0).^24^,^25^ Analysis of the crystal structure (Fig. 1B) showed key pi–pi stacking interactions between the pyridine ring and Trp128 at the outer pocket, and further pi–pi interactions between the tolyl ring and Phe268/Phe320 within the deeper, lipophilic pocket. No hydrogen bonding interactions between the ligand and the protein were observed, so it was considered that these pi–pi interactions were crucial for binding, in addition to general lipophilic interactions, as observed for the palmitoleate ligand (Fig. 1A). Notably, the orientation of Trp128 is significantly shifted in our crystal structures compared to previous structures like PDB ; 4UZQ. This shift shows a more open form of the binding site. Also in contrast to the *O*-palmitoleoyl serine structure, **3** does not interact with the oxyanion hole of the active site.

Two general synthetic methods were used to prepare new analogues: either an activated amide coupling reaction between the aminoheterocycle and the 2-phenoxyacetic acid; or a nucleophilic substitution reaction between the 2-chloroacetamide and the relevant phenoxide (Scheme 1; see, ESI‡). Inhibition of NOTUM carboxylesterase activity was routinely measured in an OPTS biochemical assay as described above. Selected compounds were then screened for NOTUM occupancy in a FP-biotin competition assay^26^ and/or inhibition of NOTUM activity in a Wnt/β-catenin signaling pathway TCF/LEF reporter (luciferase) HEK293 cell line with exogenous Wnt3a and NOTUM.^14^,^22^ Compounds were also screened for aqueous solubility, transit performance in MDCK-mdr1 cell lines for permeability, and metabolic stability in human and mouse liver microsomes (HLM and MLM resp.) as a measure of clearance.

The SARs were directed at exploring four principle areas of the original hit **3**: the 2-heteroatom linker (**4–7**), substituents on the phenoxy ring (**8–18**), the acetamide backbone (**19**, **20**) (Table 1), and the amide group (**21–53**) (Table 2). Furthermore, target compounds were designed to have molecular and physicochemical properties consistent with CNS drug-like space. It is generally accepted that CNS drugs tend to occupy a restricted property space compared to general orally available drugs,^23^ and extensive efforts have been made to define attributes that are desirable for molecules to have good brain exposure. One such model is the CNS MPO score^24^,^25^ which we routinely used in our compound design. A second useful design metric was lipophilic ligand efficiency (LLE)^27^,^28^ used to track improvements in NOTUM activity against lipophilicity (clog *P*) of the inhibitors. The palmitoleate pocket of NOTUM is a very lipophilic environment and we were keen to avoid gains in activity simply through increased compound lipophilicity.

**Table 1:** Inhibition of NOTUM activity. SAR of the heteroatom linker, the phenoxy ring and the acetamide

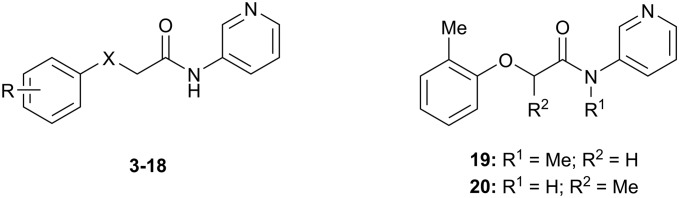			
#	X	R	IC_50_^*a*^ (μM)
**3**	O	2-Me	33
**4**	NH	2-Me	60
**5**	S	2-Me	110
**6**	CH_2_	2-Me	140
**7**	NMe	2-Me	23
**8**	O	H	150
**9**	O	2-F	290
**10**	O	2-OMe	170
**11**	O	2-CN	120
**12**	O	2-CF_3_	37
**13**	O	2-Cl	14
**14**	O	3-Cl	310
**15**	O	3-Me	37% I @ 100 μM
**16**	O	4-Me	20% I @ 100 μM
**17**	O	2-Me;4-F	20
**18**	O	2-Me;6-Me	44
**19**	—	—	180
**20**	—	—	100

^*a*^Values are geometric means of *n* = 2–8 experiments quoted to 2 s.f. Screening data only passed the quality control criteria if the screening plates demonstrated a *Z*′ > 0.5 and the NOTUM inhibition activity of the positive control was within acceptable limits (IC_50_ 0.6–1.1 nM). Differences of <2-fold should not be considered significant. See also ESI Table S1 for s.e.m.

**Table 2:** Inhibition of NOTUM activity. SAR of the amide heterocycles

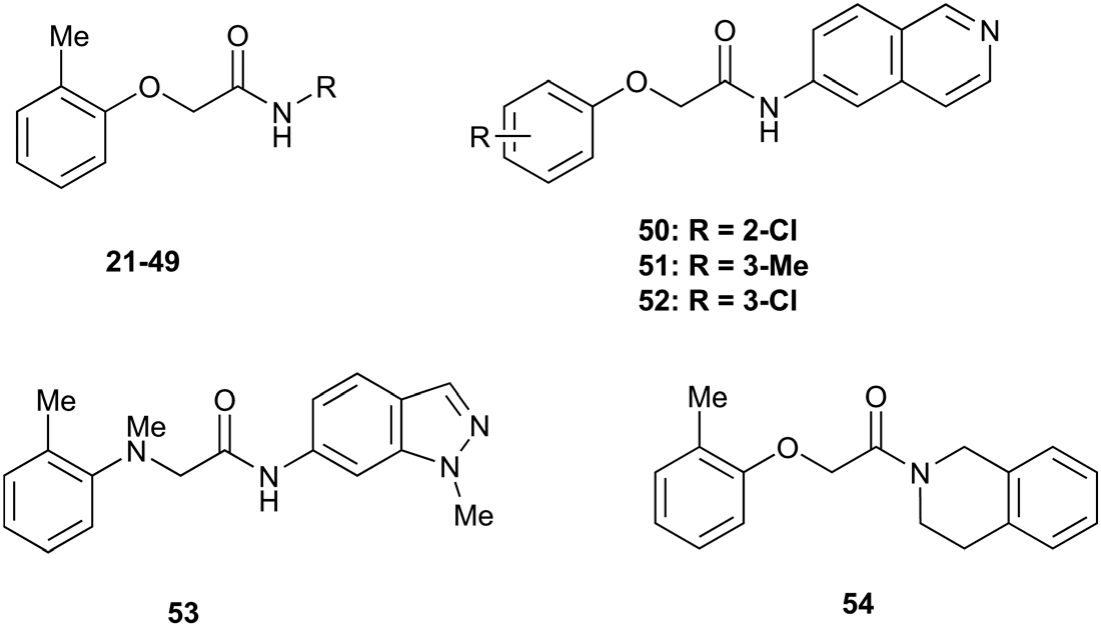					
#	R	IC_50_^*a*^ (μM)	#	R	IC_50_^*a*^ (μM)
**3**	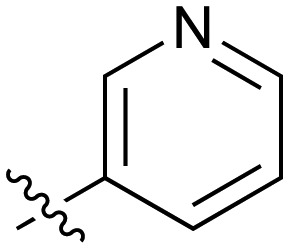	33	**21**	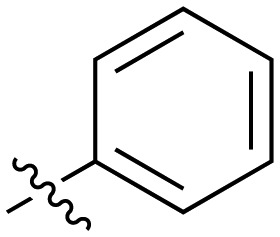	1.6
**22**	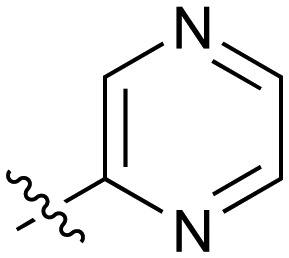	9.4	**23**	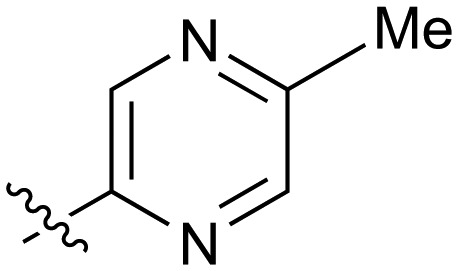	3.6
**24**	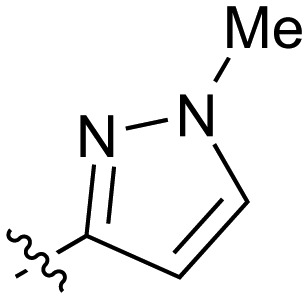	72	**25**	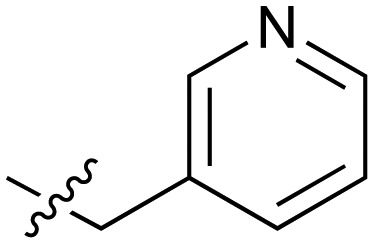	100
**26**	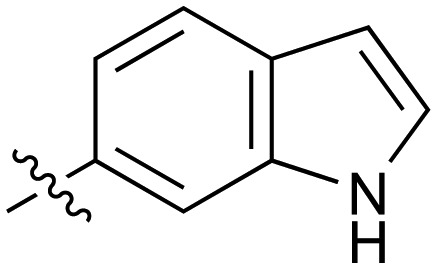	0.21	**27**	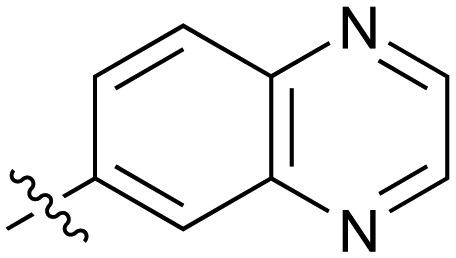	0.68
**28**	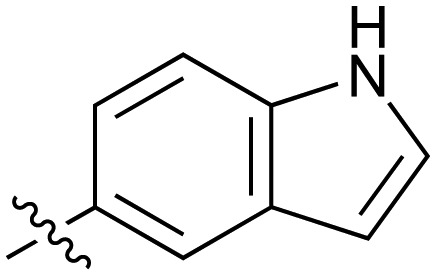	0.33	**29**	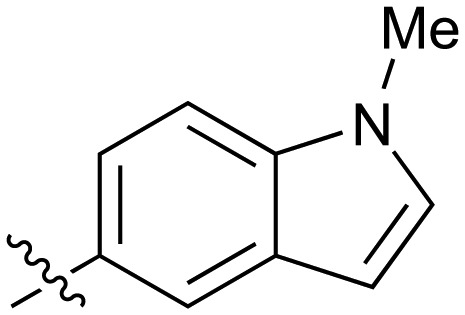	0.24
**30**	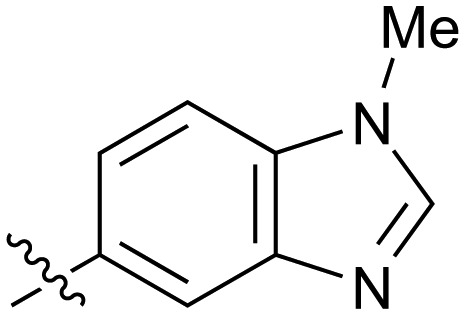	0.52	**31**	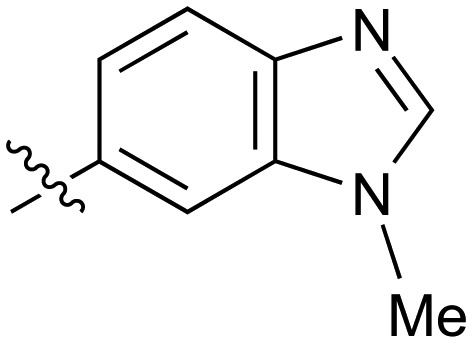	0.98
**32**	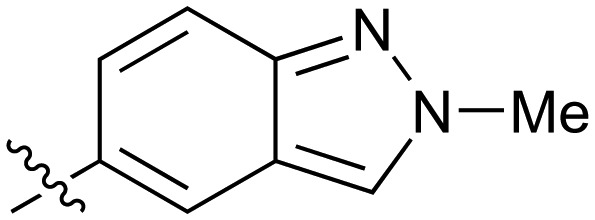	0.36	**33**	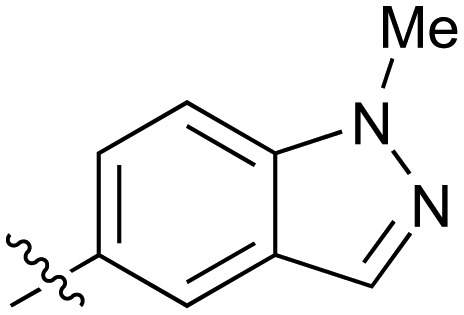	0.27
**34**	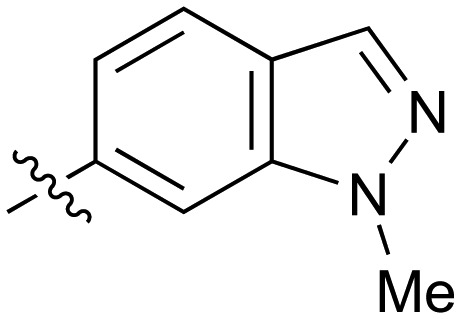	0.27	**35**	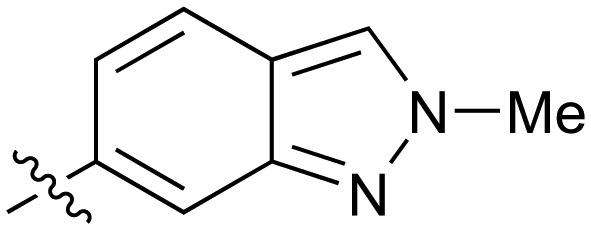	0.28
**36**	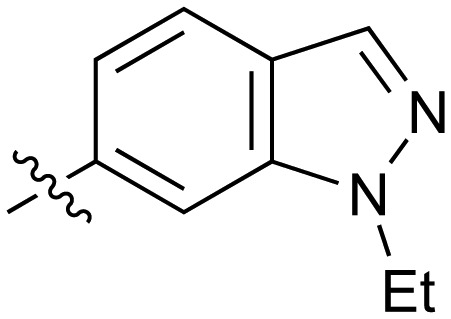	0.20	**37**	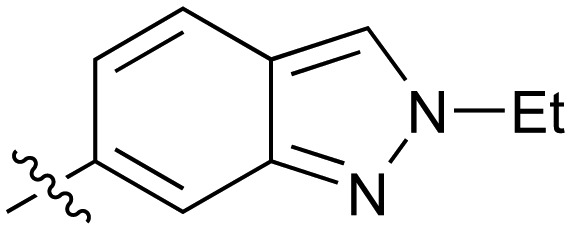	0.068
**38**	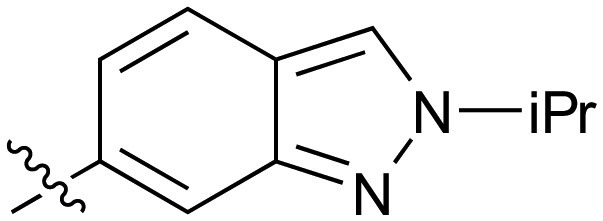	0.032	**39**	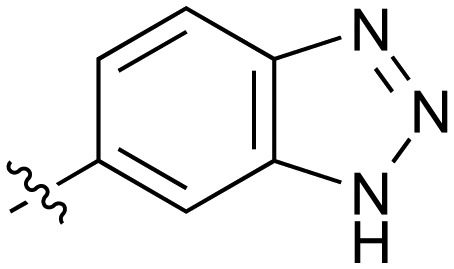	0.12
**40**	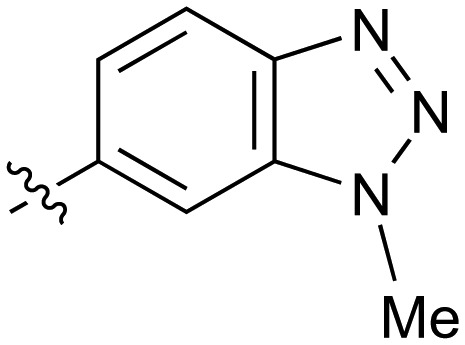	0.44	**41**	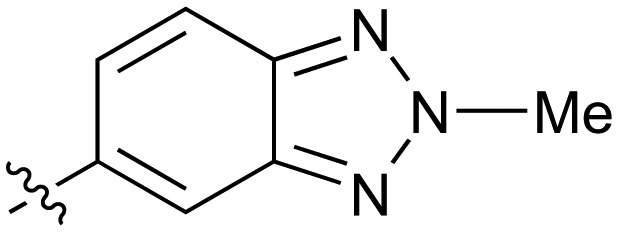	0.27
**42**	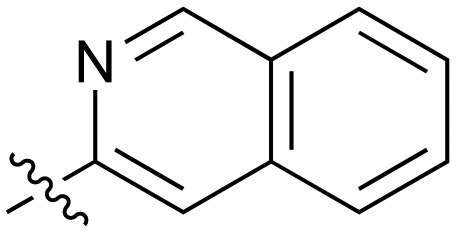	7.4	**43**	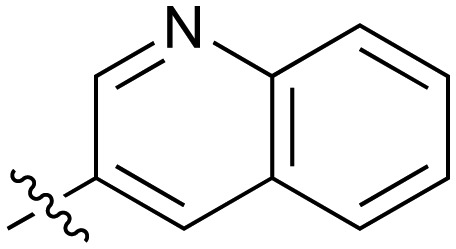	2.5
**44**	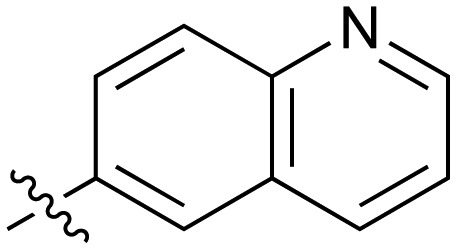	1.2	**45**	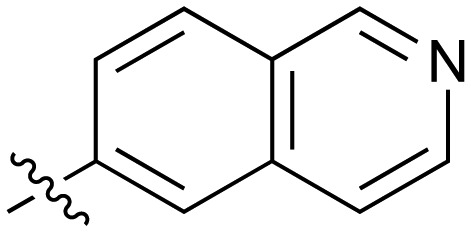	0.085
**46**	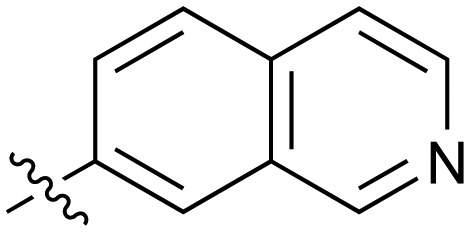	0.97	**47**	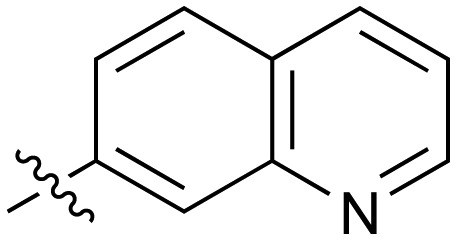	0.43
**48**	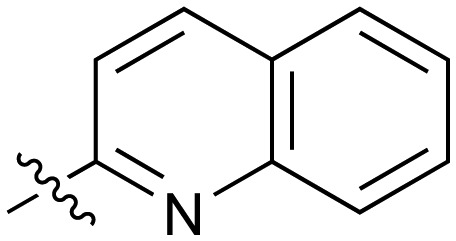	15	**49**	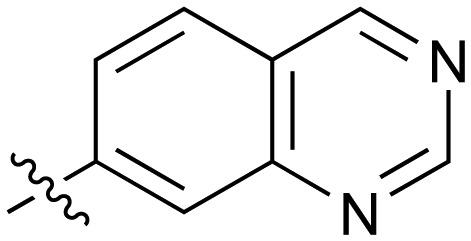	0.67
**50**	—	0.069	**51**	—	19
**52**	—	5.2	**53**	—	5.7
**54**	—	7.9			

^*a*^Values are geometric means of *n* = 2–8 experiments quoted to 2 s.f. Screening data only passed the quality control criteria if the screening plates demonstrated a *Z*′ > 0.5 and the NOTUM inhibition activity of the positive control was within acceptable limits (IC_50_ 0.6–1.1 nM). Differences of <2-fold should not be considered significant. See also ESI Table S1 for s.e.m.

Initial studies suggested that replacing the O-atom linker was not an effective strategy for increasing activity, with potency decreasing from O > NH > S > CH_2_ (**3–6**) (Table 1). The preference for an O-linker was believed to be due to a favorable internal H-bond with the amide NH holding the molecule in a low energy conformation that closely mimicked the NOTUM binding conformation. The NMe linker **7** was similar in potency for this matched pair (**3***vs.***7**) although this did not prove to be a general trend, and so the O-linker was retained for the next phase of SAR. *N*-Methylation of the amide group (**19**) or the 2-phenoxy propanamide backbone (**20**) was tolerated but offered no advantage (Table 1).

Guided by the NOTUM-**3** crystal structure, a concise investigation of SAR of the phenoxy ring indicated the original 2-methyl group in **3** was preferred with a beneficial 2-fold increase in potency only achieved by direct replacement with a 2-chlorine atom **13** (Table 1). Moving the Cl atom or Me group to the 3-, 4-positions gave a significant decrease in potency (**14**, **15**, **16**) and was not pursued further at this point. These results were consistent with the structural information showing minimal space to accommodate any group larger than H- or F-atom (**17**) in these positions. The symmetrical 2,6-diMe phenoxy ether **18** was tolerated but offered no advantage over a single 2-Me substituent. From this limited set, most groups were detrimental, or offered little advantage, compared to having no substituent (**8**; R = H) with a preference for small lipophilic groups at the 2-position of the ring such as 2-Me, 2-Cl and 2-CF_3_.

The next phase of SAR was to explore the *N*-acetamide substituent with a variety of heterocycles (Table 2). Although swapping the 3-Py group for the Ph (**21**) gave a boost in activity, this was at a significant penalty in added lipophilicity (clog *P* 2.9, LLE = 2.8), and pyrazine **22** proved to be a more efficient inhibitor (clog *P* 0.8, LLE = 4.2). Interestingly, the 3-(aminomethyl)pyridine amide **25** showed a decrease in potency from **3** suggesting that the introduction of the methylene linker disrupted the positioning of the ligand. The most significant increase in activity was achieved with benzo-fused heterocycles such as **26** and **27**. These results prompted a more systematic investigation of both 6,5- and 6,6-heterocycles.

Fused 6,5-ring systems were well tolerated, with a range of indoles (**28**, **29**), benzimidazoles (**30**, **31**), indazoles (**32–38**) and benzotriazoles (**39–41**) having NOTUM inhibition <1 μM (Table 2). With good activity now achieved within this series, indazole **34** (IC_50_ 0.27 ± 0.06 μM) (clog *P* 3.1; LLE 3.6; CNS MPO 5.6) was selected as a representative early lead for *in vitro* ADME profiling. Indazole **34** had low aqueous thermodynamic solubility (2 μg mL^–1^) and moderate cell permeability as measured by transit performance in the MDCK-mdr1 cell line with no evidence of P-gp mediated efflux (AB/BA *P*_app_ 5.1/6.6 × 10^6^ cm s^–1^). However, **34** had very poor metabolic stability in both HLM and MLM and was found to be rapidly degraded in a NADPH-independent manner (Table 3). Hydrolytic stability of **34** was tested in three buffer systems (pH 4, 7.4, 10) at multiple time points up to 18 h and only parent was detected by UPLC-MS confirming degradation of **34** was not caused by simple hydrolysis. These results highlighted the need to further improve NOTUM inhibition activity whilst simultaneously developing compounds with good metabolic stability. Hence the microsomal stability assay became a priority secondary screen (*vide infra*).

**Table 3:** *In vitro* microsomal stability data for selected compounds

#	NOTUM IC_50_ (μM)	HLM (*t*_1/2_ min)	MLM (*t*_1/2_ min)	clog *P*
**3**	33	—	1.1^*a*^	1.7
**22**	9.4	—	Not detected^*a*^	0.8
**34**	0.27	11.6^*a*^	Not detected^*a*^	3.1
**38**	0.032	5.1^*a*^	5.7^*a*^	4.0
**39**	0.12	—	1.8^*a*^	2.8
**45**	0.085	12.1^*a*^	11.0^*a*^	3.0
**50**	0.069	—	3.3^*a*^	3.3
**53**	5.7	—	5.2^*a*^	3.1
**54**	7.9	—	Not detected^*a*^	3.5

^*a*^NADPH independent metabolism.

Further investigation of SAR within the indazole series showed activity was increased by addition of a larger alkyl group (Me, Et, iPr) on the N1 or N2 position (**35***vs.***37***vs.***38**). Particularly noteworthy is the 2-isopropylindazole **38** (IC_50_ 0.032 ± 0.004 μM) (clog *P* 4.0; LLE 3.5; CNS MPO 5.1) which was the most potent NOTUM inhibitor from this 6,5-series. Benzotriazoles (**39–41**) were well tolerated with the SAR of the N1-Me (**40**) and N2-Me (**41**) derivatives tracking their counterparts in the indazole series (*e.g.***32***vs.***35***vs.***41**). The unsubstituted benzotriazole **39** (IC_50_ 0.12 ± 0.04 μM) (clog *P* 2.8; LLE 4.2; CNS MPO 5.0) offered a slight advantage and again showed that a free N1-H could be accommodated as seen previously with indole **26**. However, both **38** and **39** were also rapidly degraded in HLM and MLM in a NADPH-independent manner.

Fused 6,6-ring systems showed more variation in their NOTUM inhibition activities with the position of the N atom(s) critical for achieving good activity (Table 2). A systematic migration of a single N atom around each available position of the heterocycle (**42–48**) showed a preference for the N atom in the distal ring with a clear advantage for the 6-isoquinoline isomer **45** (IC_50_ 0.085 ± 0.011 μM) (clog *P* 3.0; LLE 4.1; CNS MPO 5.6). Multiple N atoms were accommodated in the distal ring of the 6,6-rings as shown with quinazoline **49** but was still inferior to **45**.

With good NOTUM inhibition activity achieved across a range of amides, preferred compounds (IC_50_ < 0.50 μM) were submitted for NOTUM X-ray co-crystallography studies to elucidate their binding modes. Structures of benzotriazole **39** (Fig. 1C) and isoquinoline **45** (Fig. 1D) were solved and showed that, while having similar potencies, they differed in their adopted binding conformations. Both compounds formed pi–pi stacking interactions with Trp128 through their heterocyclic rings but there was a distinct difference in the orientation of their phenoxyacetamide backbones. Benzotriazole **39** adopted a similar orientation as the pyridinyl fragment **3** (Fig. 1B). In contrast, isoquinoline **45** showed a 180° rotation around the amide bond with the carbonyl oxygen now pointing away from the oxyanion hole and the methyl group of the tolyl group occupying the opposite side of the lipophilic pocket.

An overlay of the crystal structures of **3** and **45** showed that the phenoxy ring of **45** was shifted slightly (*ca.* 1.28 Å) towards the front of the binding pocket, suggesting the possibility of additional space for further modification to this ring. Therefore, substitution on the phenoxy ring was revisited in the **45** series in an attempt to achieve optimal occupancy of the lipophilic pocket and increase potency. However, once again, only a 2-Cl group **50** (IC_50_ 0.069 ± 0.011 μM) (clog *P* 3.3; LLE 3.9; CNS MPO 5.5) was accommodated with the 3-Me **51** and 3-Cl **52** giving a significant decrease in potency, although still superior to the direct analogues in the 3-pyridinyl series (**14** and **15**). Isoquinoline amides **45** and **50** were screened in liver microsomes and also showed rapid degradation in a NADPH-independent manner.

Representative inhibitors were tested in a NOTUM occupancy assay using FP-biotin, a serine hydrolase activity-based probe, whereby labelling of NOTUM with FP-biotin can be blocked by an inhibitor occupying the active site of NOTUM (Fig. 2A). All inhibitors showed some ability to prevent labelling by FP-biotin, confirming they competitively bind to NOTUM, albeit with modest potency compared to **1**, and the rank order of binding tracks with NOTUM IC_50_ activity in the OPTS assay.

**Fig. 2:**
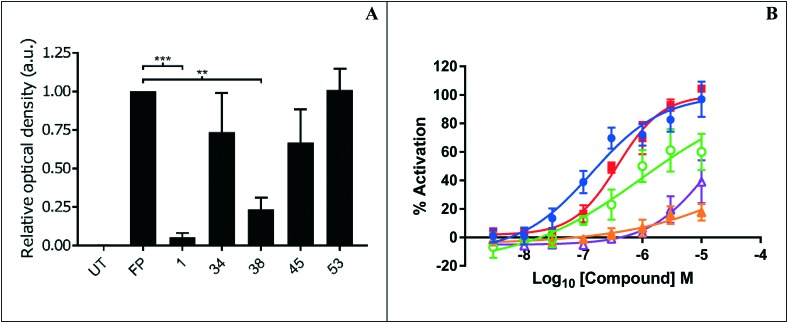
(A) NOTUM activity-based occupancy assay was performed with FP-biotin (2 μM) and test compounds (10 μM) for 10 minutes in conditioned media from HEK293S cells stably transfected with a NOTUM lentiviral construct. Relative occupancy was calculated by optical density of fluorescent band detecting the level of biotinylation of NOTUM using image studio lite 5.2, compared to the control-treated sample which was set to 1. *N* = 3 with S.D. statistical significance is calculated using an unpaired *t*-test with Welch's adjustment (***p ≤ 0.0005, **p ≤ 0.005). UT, untreated. (B) Percentage activation of the Wnt pathway *via* inhibition of NOTUM by compounds in the stable TCF-Lef SuperTOPFLASH luciferase reporter HEK293T cell line. Cells were treated in 8 point concentration response curves from 3 nM to 10 μM for 18 hours. % activation of the TCF/Lef reporter gene was calculated by normalizing data to DMSO (minimum) and to compound **1** at 10 μM (maximum) control wells. EC_50_s were calculated using a 4PL fit in Graphpad prism and Dotmatics studies. Compound key: **1** (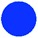), **37** (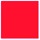), **38** (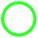), **45** (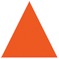), **49** (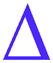).

Preferred inhibitors were then screened in the cell-based TCF/LEF reporter gene (Luciferase) assay to assess their ability to restore Wnt/β-catenin signaling when activated by exogenous Wnt3a (100 ng mL^–1^) in the presence of NOTUM (500 ng mL^–1^) (Fig. 2B). Assay validation studies with a training set of NOTUM inhibitors showed that nanomolar activity in the OPTS assay (IC_50_ < 1 μM) was required to show activity in the cell assay; these findings are consistent with previous reports.^14^,^22^ Compound **1** showed an effective activation of Wnt signaling (EC_50_ 0.138 ± 0.053 μM; *n* = 4) in this model system through inhibition of NOTUM (ESI,‡ Table S2). Compound **37** also showed activation of Wnt signaling *via* inhibition of NOTUM (EC_50_ 0.386 ± 0.199 μM; *n* = 4). Compound **38** demonstrated 60% inhibition of NOTUM at 3 μM (estimated EC_50_ 0.934 ± 0.657 μM; *n* = 4), whilst **45** and **49** showed 20% and 38% inhibition at 10 μM respectively. More stringent EC_50_ determination was precluded by bell shaped dose response curves for the compounds when tested at concentrations > 10 μM. In contrast, **27** and **35** demonstrated a supramaximal response consistent with dual activity in this assay, preventing accurate EC_50_ determination. These compounds not only inhibit NOTUM as shown by the Wnt3a-mediated activation of the reporter system but also acts as stabilizers of the luciferase enzyme giving the supramaximal response.^29^ This luciferase activity is consistent with similar chemical structures to **3** that have been reported to be inhibitors of luciferase in the PubChem database.^30^

Throughout the course of analogue preparation, preferred examples were assessed for microsomal stability (Table 3). However, the majority of the compounds tested were rapidly metabolized in an NADPH-independent manner with *t*_1/2_ < 12.5 min. One approach to mitigate this issue was to introduce a NMe linker as a direct replacement for the O linker with indazole **53** (*cf.***34**) but this led to a 25-fold decrease in activity and had no effect on alleviating the NADPH-independent metabolism. When an isolipophilic non-aromatic amide was profiled in MLM, tetrahydroisoquinoline **54** was also found to have rapid degradation in a NADPH-independent manner. Even revisiting some of the more polar analogues such as **3** and **22** in MLM suffered a similar outcome. Rapid NADPH-independent metabolism of structurally related 2-phenoxypropanamides has recently been reported suggesting a common metabolic liability associated with this type of scaffold.^31^ Hence, in light of this work, we recommend assessing the stability of *N*-aryl 2-phenoxyacetamide scaffolds to NADPH-independent metabolism in liver microsomes early in the drug discovery process.

In summary, a crystallographic fragment screen with carboxylesterase NOTUM identified 2-phenoxyacetamide **3** as binding in the palmitoleate pocket with modest inhibition activity. Optimization of hit **3** by SAR studies guided by SBDD identified indazole **38** and isoquinoline **45** as potent inhibitors of NOTUM with properties entirely consistent with CNS drug space. Through direct inhibition of NOTUM, **38** and **45** may prove to be valuable chemical tools for use in *in vitro* disease models where over activity of NOTUM suppressing Wnt signaling is an underlying cause. However, ultimately, it was not possible to combine NOTUM inhibition activity with metabolic stability in this series as the majority of the compounds tested in liver microsomes were rapidly metabolized in an NADPH-independent manner. These compounds represent a step forward in identifying new chemical matter to study the role of NOTUM in Wnt signaling, and work is ongoing to identify compounds suitable for *in vivo* studies to validate NOTUM as a target in Wnt signaling related CNS disorders.

## Funding Sources

E. Y. J.: Structural analysis was performed by Y. Z., L. V., R. R. R. and E. Y. J. supported by Cancer Research UK (Programme Grant C375/A17721) and the Wellcome Trust (PhD Training Programme 102 164/B/13/Z). The Wellcome Trust funds the Wellcome Centre for Human Genetics, University of Oxford (Centre Grant 203 141/Z/16/Z). P. V. F.: This work was supported by the Alzheimer's Research UK (ARUK) and the Francis Crick Institute. The ARUK UCL Drug Discovery Institute is core funded by Alzheimer's Research UK (520909). The Francis Crick Institute receives its core funding from Cancer Research UK (FC001002), the UK Medical Research Council (FC001002), and the Wellcome Trust (FC001002).

## Abbreviations

Aβ: Amyloid-beta
ABPP: Activity-based protein profiling
AD: Alzheimer's disease
ADME: Absorption distribution metabolism elimination
CNS: Central nervous system
FP: Fluorophosphonate
HLM: Human liver microsomes
LE: Ligand efficiency
LLE: Lipophilic ligand efficiency
MLM: Mouse liver microsomes
MPO: Multiparameter optimization
NADPH: Nicotinamide adenine dinucleotide phosphate
OPTS: Trisodium 8-octanoyloxypyrene-1,3,6-trisulfonate
P-gp: P-Glycoprotein
PTM: Post translational modifications
SAR: Structure activity relationship
SBDD: Structure based drug design
TPSA: Topological polar surface area
UPLC-MS: Ultra performance liquid chromatography – mass spectrometer

## Conflicts of interest

There are no conflicts of interest to declare.

## Supplementary Material

Supplementary information

## References

[cit1] Nusse R., Clevers H. (2017). Cell.

[cit2] Niehrs C. (2006). Oncogene.

[cit3] Niehrs C. (2012). Nat. Rev. Mol. Cell Biol..

[cit4] Malinauskas T., Jones E. Y. (2014). Curr. Opin. Struct. Biol..

[cit5] Polakis P. (2012). Cold Spring Harbor Perspect. Biol..

[cit6] Liu C.-C. (2014). Neuron.

[cit7] Marzo A. (2016). Curr. Biol..

[cit8] Zhou Y. (2014). J. Clin. Invest..

[cit9] Parr C., Mirzaei N., Christian M., Sastre M. (2015). FASEB J..

[cit10] Janda C. Y., Garcia K. C. (2015). Biochem. Soc. Trans..

[cit11] Gerlitz O., Basler K. (2002). Genes Dev..

[cit12] Kakugawa S. (2015). Nature.

[cit13] Zhang X. (2015). Dev. Cell.

[cit14] Tarver J. E. (2016). Bioorg. Med. Chem. Lett..

[cit15] Manallack D. T. (2007). Perspect. Med. Chem..

[cit16] Wager T. T., Chandrasekaran R. Y., Hou X., Troutman M. D., Verhoest P. R., Villalobos A., Will Y. (2010). ACS Chem. Neurosci..

[cit17] Manallack D. T., Prankerd R. J., Yuriev E., Oprea T. I., Chalmers D. K. (2013). Chem. Soc. Rev..

[cit18] Charifson P. S., Walters W. P. (2014). J. Med. Chem..

[cit19] Di L., Rong H., Feng B. (2013). J. Med. Chem..

[cit20] Suciu R. M., Cognetta A. B., Potter Z. E., Cravatt B. F. (2018). ACS Med. Chem. Lett..

[cit21] https://www.diamond.ac.uk/Instruments/Mx/Fragment-Screening/Fragment-Libraries.html. Accessed 18th October 2018.

[cit22] Barbosa J., Carson K. G., Gardyan M. W., He W., Lombardo V., Pabba P. and Tarver Jr J. Inhibitors of notum pectinacetylesterase and methods of their use, US20120065200, 2012.

[cit23] Rankovic Z. (2015). J. Med. Chem..

[cit24] Wager T. T. (2010). ACS Chem. Neurosci..

[cit25] Rankovic Z. (2017). J. Med. Chem..

[cit26] Liu Y., Patricelli M. P., Cravatt B. F. (1999). Proc. Natl. Acad. Sci. U. S. A..

[cit27] Leeson P. D., Springthorpe B. (2007). Nat. Rev. Drug Discovery.

[cit28] Johnson T. W., Gallego R. A., Edwards M. P. (2018). J. Med. Chem..

[cit29] Auld D. S., Thorne N., Nguyen D.-T., Inglese J. A. (2008). ACS Chem. Biol..

[cit30] Thorne N. Shen M. Lea W. A. Simeonov A. Lovell S. Auld D. S. Inglese J., Chem. Biol., 2012, 19 , 1060 –1072 , . See also: https://pubchem.ncbi.nlm.nih.gov/substance/7972821#section=Top Accessed 10^th^ December 2018 . 22921073 10.1016/j.chembiol.2012.07.015 PMC3449281

[cit31] Chacko S. (2018). J. Med. Chem..

