# Supplementary material for: Discovery of 2-phenoxyacetamides as inhibitors of the Wnt-depalmitoleating enzyme NOTUM from an X-ray fragment screen
Source: Medchemcomm. 2019 Apr 29;10(8):1361–9. doi: 10.1039/c9md00096h (PMC6727465; doi:10.1039/c9md00096h)
Supplement: Supplementary file 1 [file MD-010-C9MD00096H-s001.pdf]

Electronic Supplementary Material (ESI) for MedChemComm.
This journal is © The Royal Society of Chemistry 2019

Discovery of 2-phenoxyacetamides as inhibitors of the Wnt-depalmitoleating enzyme NOTUM from an X-ray Fragment Screen

Benjamin N. Atkinson,[a, ‡] David Steadman,[a, ‡] Yuguang Zhao,[b, ‡] James Sipthorp,[a, c] Luca Vecchia,[b] Reinis R. Ruza,[b] Fiona Jeganathan[a], Georgie Lines,[a] Sarah Frew,[a] Amy Monaghan,[a] Svend Kjær,[c] Magda Bictash,[a] E. Yvonne Jones,[b, *] Paul V. Fish.[a, c, *]

[a] Alzheimer's Research UK UCL Drug Discovery Institute, University College London, Cruciform Building, Gower Street, London, WC1E 6BT, U.K.

[b] Division of Structural Biology, Wellcome Centre for Human Genetics, University of Oxford, The Henry Wellcome Building for Genomic Medicine, Roosevelt Drive, Oxford, OX3 7BN, U.K.

[c] The Francis Crick Institute, 1 Midland Road, London, NW1 1AT, U.K.

ELECTRONIC SUPPORTING INFORMATION

Page Index:

Page S2 Chemistry
Page S19 NOTUM protein expression and purification
Page S21 OPTS biochemical assay
Page S23 FP-biotin competition assay
Page S24 TCF/LEF reporter (Luciferase) cell based assay
Page S25 In vitro ADME screens
Page S26 Protein crystallization, data collection and structure solution
Page S29 References

Chemistry

General methods

Unless preparative details are provided, all reagents were purchased from commercial suppliers and used without further purification. Solvents were of ACS reagent grade or higher and purchased from commercial suppliers without further purification. Anhydrous solvents were purchased as such from Acros Organics or Sigma Aldrich. Thin layer chromatography (TLC) was carried out on aluminium backed silica plates. The plates were visualized under UV (254 nm) light, followed by staining with phosphomolybdic acid dip or potassium permanganate and gentle heating. Compound purification by column chromatography was performed using a Biotage Isolera using prepacked Biotage SNAP KP-Sil silica cartridges or Biotage SNAP Ultra C18 reverse phase cartridges. Organic solvent layers were routinely dried with anhydrous $MgSO_4$ and concentrated using a Büchi rotary evaporator. ^{1}H NMR / ^{13}C NMR spectra were run in deuterated (≥99.5%) solvents on either a Bruker Avance 300 (300 MHz), Bruker Avance 400 (400 MHz), Bruker Avance 600 (600 MHz) or Bruker Avance 700 (700 MHz). Chemical shifts (δ) are reported as parts per million (ppm), coupling constants (J) are reported in Hz and signal multiplicities are reported as singlet (s), doublet (d), triplet (t), quartet (q), pentet (p), quintet (qu), sextet (sext), doublet of doublets (dd), doublet of triplets (dt), triplet of triplets (tt), multiplet (m), or broad singlet (br s). LCMS analysis was performed on a Waters Acquity H-Class UPLC system with either an acidic (HSS C18 Column, H_2O:acetonitrile, 0.1% TFA) or basic (BEH C18 Column, H_2O:acetonitrile, 10 mM NH_4OH) mobile phase. HRMS data acquisition was on a Waters Micromass LCT Premier electrospray time-of-flight (ESI-TOF) mass spectrometer. The observed mass and isotope pattern matched the corresponding theoretical values as calculated from the expected elemental formula.

Compound purity

Purity of screening compounds **3**-**54** was evaluated by NMR spectroscopy and LCMS analysis. All compounds had purity ≥ 95 %.

Abbreviations

DMF, *N,N*-dimethylacetamide; DMSO, dimethylsulfoxide; RT, room temperature; TFA, trifluoroacetic acid.

General amide coupling procedure – Procedure A

N,N-Diisopropylethylamine (1.4 eq) was added to a solution of *O*-(1*H*-benzotriazol-1-yl)-*N,N,N',N'*-tetramethyluronium hexafluorophosphate (HBTU) (1.1 eq) and desired acid (1.0 eq) in DMF (3 mL) at 0 °C (external). The mixture was then allowed to warm to RT before addition of amine (1.0 eq). The reaction mixture was stirred at RT for 16 h and the resulting crude material was purified by reverse phase chromatography (0-100 %, MeCN:H_2O, 10 mM NH_4OH modifier). The desired fractions were then concentrated under reduced pressure to give the amide product. A few compounds required additional purification by normal phase chromatography (see text for conditions).

3 2-(2-Methylphenoxy)-*N*-(3-pyridyl)acetamide

Prepared by procedure A with 2-methylphenoxyacetic acid (124 mg, 0.75 mmol) and 3-aminopyridine (70 mg, 0.75 mmol). Isolated as an-off white solid (79 mg, 59 %).

^{1}H NMR (600 MHz, DMSO-d_6) δ 10.26 (s, 1H), 8.78 (d, *J* = 2.3 Hz, 1H), 8.29 (dd, J_{HH} = 4.7, 1.4 Hz, 1H), 8.06 (ddd, *J* = 8.3, 2.3, 1.4 Hz, 1H), 7.37 (dd, *J* = 8.3, 4.7 Hz, 1H), 7.20 – 7.11 (m, 2H), 6.88 (dd, *J* = 7.5, 5.8 Hz, 2H), 4.75 (s, 2H), 2.26 (s, 3H).

^{13}C NMR (151 MHz, DMSO-d_6) δ 167.4, 156.0, 144.6, 141.3, 135.1, 130.6, 126.9, 126.7, 126.2, 123.6, 121.0, 111.6, 67.3, 16.1.

LCMS (Acidic method) Rt 1.59 mins, MS m/z 243.3 [M+H]+

HRMS (m/z TOF MS ES^+) for $C_{14}H_{14}N_2O_2$ $[M+H]^+$ calc. 243.1128, observed 243.1130.

4 2-(2-Methylanilino)-*N*-(3-pyridyl)acetamide

In a 20 mL microwave vial, *o*-toluidine (0.070 mL, 0.66 mmol), potassium iodide (78 mg, 0.47 mmol) and potassium carbonate (97 mg, 0.70 mmol) were dissolved in *N,N*-dimethylformamide (2.5 mL) and stirred for 30 minutes. To this solution 2-chloro-*N*-(3-pyridyl)acetamide (80 mg, 0.47 mmol) was added and the reaction was sealed and heated to 70 °C for 4 h. After this time, product formation was observed, so the reaction was concentrated and purified by flash silica chromatography (0-10% MeOH:CH_2Cl_2). Product fractions were identified by LCMS and TLC analysis, and then concentrated to give an orange oil (21 mg, 19 %).

^{1}H NMR (600 MHz, methanol-d_4) δ 8.73 (d, *J* = 2.4 Hz, 1H), 8.26 (dd, *J* = 4.8, 1.5 Hz, 1H), 8.11 (ddd, *J* = 8.4, 2.4, 1.5 Hz, 1H), 7.38 (dd, *J* = 8.4, 4.8 Hz, 1H), 7.14 – 6.89 (m, 2H), 6.64 (t, *J* = 7.3 Hz, 1H), 6.56 – 6.40 (m, 1H), 4.00 (s, 2H), 2.24 (s, 3H).

^{13}C NMR (151 MHz, methanol-d_4) δ 172.93, 146.98, 145.42, 142.21, 136.95, 131.18, 129.39, 128.02, 125.27, 124.08, 118.99, 110.88, 49.56, 17.61.

LCMS (Acidic method) Rt 1.30 mins, MS m/z 242.2 [M+H]+

5 2-(*o*-Tolylsulfanyl)-*N*-(3-pyridyl)acetamide

Prepared by procedure A with *o*-tolylsulfanylacetic acid (80 mg, 0.44 mmol) and 3-aminopyridine (42 mg, 0.45 mmol). Product was further purified by flash silica chromatography (0-7% MeOH:CH_2Cl_2). Isolated as a white solid (51 mg, 45 %).

^{1}H NMR (600 MHz, $CDCl_3$) δ 8.68 – 8.46 (m, 2H), 8.36 (dd, *J* = 4.7, 1.2 Hz, 1H), 8.13 – 8.07 (m, 1H), 7.29 – 7.13 (m, 5H), 3.78 (s, 2H), 2.45 (s, 3H).

^{13}C NMR (151 MHz, $CDCl_3$) δ 166.88, 145.95, 141.41, 137.45, 134.17, 133.14, 130.84, 127.35, 127.29, 127.15, 123.77, 37.71, 20.40.

LCMS (Basic method) Rt 1.50 mins, MS m/z 259.1 [M+H]+

6 3-(*o*-Tolyl)-*N*-(3-pyridyl)propanamide

Prepared by procedure A with 3-(2-methylphenyl)propionic acid (80 mg, 0.49 mmol) and 3-aminopyridine (46 mg, 0.50 mmol). Isolated as a white solid (120 mg, 68 %).

^{1}H NMR (600 MHz, $CDCl_3$) δ 8.41 (d, *J* = 2.4 Hz, 1H), 8.31 (d, *J* = 4.1 Hz, 1H), 8.13 (d, *J* = 8.2 Hz, 1H), 7.48 (s, 1H), 7.27 – 7.23 (m, 1H), 7.18 – 7.12 (m, 4H), 3.06 (t, *J* = 7.7 Hz, 2H), 2.66 (t, *J* = 7.7 Hz, 2H), 2.32 (d, *J* = 10.6 Hz, 3H).

^{13}C NMR (151 MHz, $CDCl_3$) δ 171.21, 145.32, 141.15, 138.59, 136.12, 134.83, 130.64, 128.84, 127.42, 126.79, 126.42, 123.84, 38.09, 28.80, 19.42.

LCMS (Basic method) Rt 1.59 mins, MS m/z 241.2 [M+H]+

7 2-(*N*,2-Dimethylanilino)-*N*-(3-pyridyl)acetamide

In a 20 mL microwave vial, 2-chloro-*N*-(3-pyridyl)acetamide (80 mg, 0.47 mmol), potassium iodide (77 mg, 0.47 mmol) and potassium carbonate (97 mg, 0.70 mmol) were dissolved in *N,N*-dimethylformamide (2.5 mL) and stirred for 15 minutes. To this solution, *N*,2-dimethylaniline (0.06 mL, 0.47 mmol) was added and the reaction was sealed and heated to 70 ^{o}C for 6 h. After this time, product formation was observed, so the reaction was concentrated and purified by reverse phase flash silica chromatography (15-75% MeCN:H_2O, 0.1% formic acid). TLC analysis showed that the starting aniline had co-eluted with product, so further purification was carried out (0-10% MeOH:CH_2Cl_2). Product fractions were identified by LCMS and TLC analysis, and then concentrated to give a clear oil (10 mg, 8%).

^{1}H NMR (600 MHz, MeOD) δ 8.76 (d, *J* = 2.3 Hz, 1H), 8.31 – 8.23 (m, 1H), 8.17 – 8.09 (m, 1H), 7.43 – 7.37 (m, 1H), 7.21 – 7.12 (m, 3H), 6.98 (td, *J* = 7.2, 1.7 Hz, 1H), 3.73 (s, 2H), 2.81 (s, 3H), 2.40 (s, 3H).

^{13}C NMR (151 MHz, MeOD) δ 171.94, 152.32, 145.53, 142.29, 136.80, 133.52, 132.43, 129.45, 127.79, 125.29, 124.99, 121.02, 61.75, 43.08, 18.59.

LCMS (Acidic method) Rt 1.38 mins, MS m/z 256.2 [M+H]+

8 2-Phenoxy-*N*-(pyridin-3-yl)acetamide

Commercially available, purchased from Sigma Aldrich (ref: R715301-1EA)

9 2-(2-Fluorophenoxy)-*N*-(3-pyridyl)acetamide

Prepared by procedure A with 2-fluorophenoxyacetic acid (100 mg, 0.59 mmol) and 3-aminopyridine (57 mg, 0.59 mmol).). Isolated as a light pink solid (89 mg, 62 %).

^{1}H NMR (700 MHz, $CDCl_3$) δ 8.70 (d, *J* = 2.6 Hz, 1H), 8.54 (s, 1H), 8.41 (dd, *J* = 4.7, 1.5 Hz, 1H), 8.20 (ddd, *J* = 8.3, 2.6, 1.5 Hz, 1H), 7.31 (dd, *J* = 8.3, 4.7 Hz, 1H), 7.20 – 7.10 (m, 2H), 7.08 – 7.02 (m, 2H), 4.69 (s, 2H).

^{13}C NMR (176 MHz, $CDCl_3$) δ 166.53, 152.95 (d, *J* = 245.8 Hz), 146.16, 145.29 (d, *J* = 10.9 Hz), 141.67, 133.78, 127.40, 125.02 (d, *J* = 4.0 Hz), 123.84, 123.74 (d, *J* = 7.0 Hz), 116.96 (d, *J* = 18.1 Hz), 116.57, 69.36.

^{19}F NMR (659 MHz, $CDCl_3$) δ -133.95.

LCMS (Basic method) Rt 1.51 mins, MS m/z 247.1 [M+H]+

10 2-(2-Methoxyphenoxy)-*N*-(3-pyridyl)acetamide

Prepared by procedure A with 2-methoxyphenoxyacetic acid (100 mg, 0.55 mmol) and 3-aminopyridine (57 mg, 0.59 mmol). Isolated as a white solid (104 mg, 73 %).

^{1}H NMR (700 MHz, $CDCl_3$) δ 9.15 (s, 1H), 8.62 (d, *J* = 2.6 Hz, 1H), 8.38 (dd, *J* = 4.7, 1.4 Hz, 1H), 8.27 (ddd, *J* = 8.3, 2.6, 1.4 Hz, 1H), 7.30 (dd, *J* = 8.3, 4.7 Hz, 1H), 7.08 (ddd, *J* = 8.3, 7.4, 1.4 Hz, 1H), 7.01 (dd, *J* = 7.9, 1.4 Hz, 1H), 6.99 – 6.94 (m, 2H), 4.68 (s, 2H), 3.96 (s, 3H).

^{13}C NMR (176 MHz, $CDCl_3$) δ 167.83, 150.05, 147.43, 145.79, 141.34, 134.28, 127.11, 124.14, 123.88, 121.67, 117.24, 112.43, 70.92, 56.16.

LCMS (Basic method) Rt 1.51 mins, MS m/z 259.2 [M+H]+

11 2-(2-Cyanophenoxy)-*N*-(3-pyridyl)acetamide

Prepared by procedure A with 2-cyanoyphenoxyacetic acid (100 mg, 0.55 mmol) and 3-aminopyridine (58 mg, 0.62 mmol)). Isolated as a white solid (44 mg, 31 %).

^{1}H NMR (700 MHz, $CDCl_3$) δ 8.88 (d, *J* = 2.5 Hz, 1H), 8.67 (s, 1H), 8.42 (dd, *J* = 4.7, 1.5 Hz, 1H), 8.13 (ddd, *J* = 8.3, 2.5, 1.5 Hz, 1H), 7.70 – 7.57 (m, 2H), 7.31 (dd, *J* = 8.3, 4.7 Hz, 1H), 7.18 (td, *J* = 7.6, 0.6 Hz, 1H), 7.03 (d, *J* = 8.3 Hz, 1H), 4.75 (s, 2H).

^{13}C NMR (176 MHz, $CDCl_3$) δ 165.20, 158.73, 146.28, 141.74, 135.06, 133.80, 133.67, 127.17, 123.81, 123.01, 116.36, 113.05, 102.89, 67.93.

LCMS (Basic method) Rt 1.47 mins, MS m/z 254.1 [M+H]+

12 *N*-(3-Pyridyl)-2-[2-(trifluoromethyl)phenoxy]acetamide

In a 20 mL microwave vial, 2-(trifluoromethyl)phenol (106 mg, 0.66 mmol), potassium iodide (78 mg, 0.47 mmol) and potassium carbonate (97 mg, 0.70 mmol) were dissolved in *N,N*-dimethylformamide (3 mL) and stirred for 30 minutes. To this solution, 2-chloro-*N*-(3-pyridyl)acetamide (80 mg, 0.47 mmol) was added and the reaction was sealed and heated to 70 °C for 24 h. The reaction was cooled to room temperature and solvent removed *in vacuo*. The dry residue was taken up in EtOAc (50 ml), washed with water (20 mL) and separated aqueous phase was washed with EtOAc (20 mL). The combined organics were washed with saturated brine (2 x 10 ml), dried (MgSO4) and concentrating to dryness. The crude product was then purified by normal phase chromatography (10-80 %, EtOAc:CH_2Cl_2) to give the product as a white solid (81 mg, 58 %).

^{1}H NMR (700 MHz, $CDCl_3$) δ 8.70 (d, *J* = 2.5 Hz, 1H), 8.55 (s, 1H), 8.42 (dd, *J* = 4.7, 1.4 Hz, 1H), 8.17 (ddd, *J* = 8.3, 2.5, 1.4 Hz, 1H), 7.72 – 7.66 (m, 1H), 7.64 – 7.55 (m, 1H), 7.32 (dd, *J* = 8.3, 4.7 Hz, 1H), 7.17 (t, *J* = 7.6 Hz, 1H), 7.03 (d, *J* = 8.3 Hz, 1H), 4.72 (s, 2H).

^{13}C NMR (176 MHz, $CDCl_3$) δ 165.67, 146.13, 141.39, 134.00 (d, *J* = 41.6 Hz), 127.72 (q, *J* = 5.0 Hz), 126.95, 123.96 (q, *J* = 272.3 Hz), 123.86, 122.21, 118.99 (q, *J* = 30.8 Hz). 113.15, 67.45.

^{19}F NMR (659 MHz, $CDCl_3$) δ -61.68.

LCMS (Basic method) Rt 1.67 mins, MS m/z 297.1 [M+H]+

13 2-(2-Chlorophenoxy)-*N*-(3-pyridyl)acetamide

Prepared by procedure A with 2-chlorophenoxyacetic acid (100 mg, 0.54 mmol) and 3-aminopyridine (55 mg, 0.59 mmol). Isolated as a white solid (68 mg, 48 %).

^{1}H NMR (600 MHz, $CDCl_3$) δ 8.87 – 8.67 (m, 2H), 8.42 (dd, *J* = 4.7, 1.5 Hz, 1H), 8.23 (ddd, *J* = 8.3, 2.3, 1.5 Hz, 1H), 7.46 (dd, *J* = 7.9, 1.5 Hz, 1H), 7.31 (ddd, *J* = 9.0, 8.3, 3.1 Hz, 2H), 7.05 (td, *J* = 7.9, 1.2 Hz, 1H), 6.98 (dd, *J* = 8.3, 1.2 Hz, 1H), 4.69 (s, 2H).

^{13}C NMR (151 MHz, $CDCl_3$) δ 166.23, 152.59, 146.12, 141.50, 133.85, 130.69, 128.46, 127.15, 123.87, 123.58, 123.14, 114.45, 68.38.

LCMS (Basic method) Rt 1.57 mins, MS m/z 263.2 [M+H]+

14 2-(3-Chlorophenoxy)-*N*-(3-pyridyl)acetamide

Prepared by procedure A with 3-chlorophenoxyacetic acid (100 mg, 0.54 mmol) and 3-aminopyridine (55 mg, 0.59 mmol). Isolated as a white solid (73 mg, 52 %).

^{1}H NMR (600 MHz, $CDCl_3$) δ 8.66 (d, *J* = 2.5 Hz, 1H), 8.42 (dd, *J* = 4.7, 1.3 Hz, 1H), 8.31 (s, 1H), 8.22 (ddd, *J* = 8.3, 2.3, 1.3 Hz, 1H), 7.35 – 7.27 (m, 2H), 7.07 (dd, *J* = 8.3, 1.3 Hz, 1H), 7.03 (t, *J* = 2.3 Hz, 1H), 6.90 (dd, *J* = 8.3, 2.3 Hz, 1H), 4.64 (s, 2H).

^{13}C NMR (151 MHz, $CDCl_3$) δ 166.29, 157.48, 146.26, 141.62, 135.56, 133.62, 130.91, 127.55, 123.88, 123.07, 115.76, 113.05, 67.66.

LCMS (Basic method) Rt 1.61 mins, MS m/z 263.1 [M+H]+

15 2-(3-Methylphenoxy)-*N*-(3-pyridyl)acetamide

Prepared by procedure A with 3-methylphenoxyacetic acid (100 mg, 0.60 mmol) and 3-aminopyridine (56 mg, 0.60 mmol). Product isolated as a white solid (110 mg, 75 %).

^{1}H NMR (600 MHz, $CDCl_3$) δ 8.65 (d, *J* = 2.5 Hz, 1H), 8.40 (dd, *J* = 4.7, 1.4 Hz, 1H), 8.35 (s, 1H), 8.25 – 8.18 (m, 1H), 7.31 (dd, *J* = 8.3, 4.7 Hz, 1H), 7.23 (t, *J* = 7.9 Hz, 1H), 6.89 (dd, *J* = 7.9, 0.6 Hz, 1H), 6.82 (s, 1H), 6.79 (dd, *J* = 8.3, 2.5 Hz, 1H), 4.63 (s, 2H), 2.37 (s, 3H).

^{13}C NMR (151 MHz, $CDCl_3$) δ 167.10, 156.99, 146.11, 141.61, 140.37, 133.79, 129.83, 127.44, 123.83, 123.63, 115.80, 111.80, 67.64, 21.64.

LCMS (Basic method) Rt 1.61 mins, MS m/z 243.2 [M+H]+

16 2-(4-Methylphenoxy)-*N*-(3-pyridyl)acetamide

Prepared by procedure A with 4-methylphenoxyacetic acid (100 mg, 0.60 mmol) and 3-aminopyridine (56 mg, 0.60 mmol). Product isolated as a white solid (110 mg, 75 %).

^{1}H NMR (600 MHz, $CDCl_3$) δ 8.64 (d, *J* = 2.4 Hz, 1H), 8.40 (dd, *J* = 4.7, 1.5 Hz, 1H), 8.35 (s, 1H), 8.21 (ddd, *J* = 8.3, 2.4, 1.5 Hz, 1H), 7.30 (dd, *J* = 8.3, 4.7 Hz, 1H), 7.15 (dd, *J* = 9.8, 1.5 Hz, 2H), 6.93 – 6.85 (m, 2H), 4.61 (s, 2H), 2.32 (s, 3H).

^{13}C NMR (151 MHz, $CDCl_3$) δ 167.19, 154.92, 146.09, 141.60, 133.80, 132.26, 130.52, 127.44, 123.83, 114.83, 67.92, 20.64.

LCMS (Acidic method) Rt 1.45 mins, MS m/z 243.2 [M+H]+

17 2-(4-Fluoro-2-methylphenoxy)-*N*-(3-pyridyl)acetamide

In a 20 mL microwave vial, 4-fluoro-2-methylphenol (77 mg, 0.61 mmol), potassium iodide (78 mg, 0.47 mmol) and potassium carbonate (97 mg, 0.70 mmol) were dissolved in *N,N*-dimethylformamide (3 mL) and stirred for 30 minutes. To this solution, 2-chloro-*N*-(3-pyridyl)acetamide (80 mg, 0.47 mmol) was added and the reaction was sealed and heated to 70 °C for 24 h. The reaction was cooled to room temperature and solvent removed *in vacuo*. The dry residue was taken up in EtOAc (50 ml), washed with water (20 mL) and separated aqueous phase was washed with EtOAc (20 mL). The combined organics were washed with saturated brine (2 x 10 ml), dried ($MgSO_4$) and concentrating to dryness. The crude product was then purified by normal phase chromatography (10-80 %, EtOAc:CH_2Cl_2) to give the product as a white solid (53 mg, 43 % yield).

^1H NMR (700 MHz, $CDCl_3$) δ 8.63 (d, *J* = 2.6 Hz, 1H), 8.43 – 8.27 (m, 2H), 8.23 (ddd, *J* = 8.3, 2.6, 1.5 Hz, 1H), 7.32 (dd, *J* = 8.3, 4.7 Hz, 1H), 6.95 (ddd, *J* = 8.9, 3.0, 0.4 Hz, 1H), 6.88 (ddd, *J* = 8.3, 3.0, 1.5 Hz, 1H), 6.79 (dd, *J* = 8.9, 4.7 Hz, 1H), 4.60 (s, 2H), 2.36 (s, 3H).

^{13}C NMR (176 MHz, $CDCl_3$) δ 166.93, 158.08 (d, *J* = 240.9 Hz), 151.39 (d, *J* = 2.3 Hz), 146.20, 141.47, 133.74, 128.87 (d, *J* = 7.8 Hz), 127.35, 123.92, 118.21 (d, *J* = 23.1 Hz), 113.52 (d, *J* = 8.7 Hz), 113.39 (d, *J* = 23.0 Hz), 68.71, 16.63.

^{19}F NMR (659 MHz, $CDCl_3$) δ -121.34.

LCMS (Basic method) Rt 1.61 mins, MS m/z 261.1 [M+H]+

18 2-(2,6-Dimethylphenoxy)-*N*-(3-pyridyl)acetamide

Prepared by procedure A with 2,6-dimethylphenoxyacetic acid (100 mg, 0.55 mmol) and 3-aminopyridine (53 mg, 0.57 mmol). The product was then further purified by normal phase chromatography (10-100 %, EtOAc:cyclohexane) to give the product as a white solid (63 mg, 44 %).

^1H NMR (700 MHz, $CDCl_3$) δ 8.72 (t, *J* = 3.6 Hz, 2H), 8.42 (dd, *J* = 4.7, 1.3 Hz, 1H), 8.28 (ddd, *J* = 8.3, 2.4, 1.3 Hz, 1H), 7.33 (dd, *J* = 8.3, 4.7 Hz, 1H), 7.07 (d, *J* = 7.5 Hz, 2H), 7.03 – 6.96 (m, 1H), 4.44 (s, 2H), 2.32 (s, 6H).

^{13}C NMR (176 MHz, $CDCl_3$) δ 167.42, 154.07, 146.04, 141.47, 134.00, 130.45, 129.51, 129.50, 129.48, 127.24, 125.29, 123.90, 70.51, 16.52, 16.51, 16.49, 16.47.

LCMS (Basic method) Rt 1.65 mins, MS m/z 257.2 [M+H]+

19 *N*-Methyl-2-(2-methylphenoxy)-*N*-(3-pyridyl)acetamide

Prepared by procedure A with 2-methylphenoxyacetic acid (100 mg, 0.60 mmol) and 3-(methylamino)pyridine (66 mg, 0.60 mmol). Product isolated as a white solid (67 mg, 43 %).

^{1}H NMR (600 MHz, $CDCl_3$) δ 8.70 – 8.42 (m, 2H), 7.55 (t, *J* = 24.6 Hz, 1H), 7.35 (dd, *J* = 8.0, 4.8 Hz, 1H), 7.18 – 7.01 (m, 2H), 6.88 (dt, *J* = 14.1, 7.2 Hz, 1H), 6.72 – 6.54 (m, 1H), 4.51 (s, 2H), 3.34 (s, 3H), 2.03 (s, 3H).

^{13}C NMR (151 MHz, $CDCl_3$) δ 166.52, 155.38, 144.20, 141.12, 135.02, 132.71, 131.40, 127.50, 126.66, 122.40, 120.64, 115.21, 112.04, 101.49, 67.94, 31.32, 16.55.

LCMS (Basic method) Rt 1.58 mins, MS m/z 257.2 [M+H]+

20 (±)-2-(2-Methylphenoxy)-*N*-(3-pyridyl)propanamide

Prepared by procedure A with (±)-2-(2-methylphenoxy)propanoic acid (80 mg, 0.44 mmol) and 3-aminopyridine (42 mg, 0.45 mmol). Product isolated as a colourless gel (106 mg, 93 %).

^{1}H NMR (700 MHz, DMSO) δ 10.28 (s, 1H), 8.77 (d, J= 2.5 Hz, 1H), 8.28 (dd, J= 4.7, 1.3 Hz, 1H), 8.07 – 8.03 (m, 1H), 7.35 (dd, J= 8.3, 4.7 Hz, 1H), 7.17 (d, J= 7.1 Hz, 1H), 7.12 (dd, J= 11.0, 4.7 Hz, 1H), 6.86 (dd, J= 14.7, 7.7 Hz, 2H), 4.87 (q, J= 6.6 Hz, 1H), 2.24 (s, 3H), 1.57 (d, J= 6.6 Hz, 3H)

^{13}C NMR (176 MHz, DMSO) δ 170.76, 155.48, 144.66, 141.33, 135.12, 130.74, 126.89, 126.73, 126.67, 123.59, 121.06, 112.39, 73.96, 18.62, 16.16

LCMS (Basic method) Rt 1.69 mins, MS m/z 257.2 [M+H]+

21 2-(2-Methylphenoxy)-*N*-phenylacetamide

Prepared by procedure A with 2-methylphenoxyacetic acid (100 mg, 0.60 mmol) and aniline (0.06 mL, 0.61 mmol). Product was isolated as a white solid (121 mg, 83 %).

^{1}H NMR (600 MHz, $CDCl_3$) δ 8.35 (s, 1H), 7.60 (d, *J* = 7.8 Hz, 2H), 7.38 (t, *J* = 7.8 Hz, 2H), 7.20 m, 3H), 6.99 (t, *J* = 7.4 Hz, 1H), 6.86 (d, *J* = 8.0 Hz, 1H), 4.63 (s, 2H), 2.38 (s, 3H).

^{13}C NMR (151 MHz, $CDCl_3$) δ 166.52, 155.32, 136.96, 131.35, 129.29, 127.50, 126.60, 125.01, 122.34, 120.07, 111.97, 67.83, 16.56.

LCMS (Basic method) Rt 1.79 mins, MS m/z 242.1 [M+H]+

22 2-(2-Methylphenoxy)-*N*-(pyrazin-2-yl)acetamide

Prepared by procedure A with 2-methylphenoxyacetic acid (100 mg, 0.60 mmol) and 2-aminopyrazine (63 mg, 0.63 mmol). Product isolated as an off-white solid (73 mg, 50 %).

^{1}H NMR (600 MHz, $CDCl_3$) δ 9.63 (d, *J* = 1.6 Hz, 1H), 8.99 – 8.80 (m, 1H), 8.41 (d, *J* = 2.5 Hz, 1H), 8.30 (dd, *J* = 2.5, 1.6 Hz, 1H), 7.22 (m, 2H), 6.99 (t, *J* = 7.2 Hz, 1H), 6.85 (d, *J* = 8.1 Hz, 1H), 4.69 (s, 2H), 2.40 (s, 3H).

^{13}C NMR (151 MHz, $CDCl_3$) δ 167.14, 155.16, 147.33, 142.42, 141.06, 137.12, 131.47, 127.42, 126.86, 122.52, 111.80, 67.64, 16.59.

HRMS (m/z TOF MS ES^+) for $C_{13}H_{13}N_3O_2$ $[M+H]^+$ calc. 244.1081, observed 244.1082.

LCMS (Basic method) Rt 1.65 mins, MS m/z 244.2 [M+H]+

23 2-(2-Methylphenoxy)-*N*-(5-methylpyrazin-2-yl)acetamide

Prepared by procedure A with 2-methylphenoxyacetic acid (100 mg, 0.60 mmol) and 2-Amino-5-methylpyrazine (67 mg, 0.61 mmol). Product isolated as a white solid (63 mg, 41 %).

^{1}H NMR (600 MHz, $CDCl_3$) δ 9.49 (d, *J* = 0.9 Hz, 1H), 8.82 (s, 1H), 8.16 (s, 1H), 7.21 (m, 2H), 6.99 (t, *J* = 7.4 Hz, 1H), 6.84 (d, *J* = 8.1 Hz, 1H), 4.67 (s, 2H), 2.56 (s, 3H), 2.39 (s, 3H).

^{13}C NMR (151 MHz, $CDCl_3$) δ 166.95, 155.22, 150.00, 144.96, 141.72, 135.79, 131.43, 127.39, 126.87, 122.45, 111.81, 67.66, 21.07, 16.60.

LCMS (Basic method) Rt 1.59 mins, MS m/z 259.1 [M+H]+

24 2-(2-Methylphenoxy)-*N*-(1-methylpyrazol-3-yl)acetamide

Prepared by procedure A with 2-methylphenoxyacetic acid (100 mg, 0.60 mmol) and 1-methylpyrazol-3-amine (64 mg, 0.66 mmol). Product isolated as a white solid (87 mg, 59 %).

^{1}H NMR (600 MHz, $CDCl_3$) δ 8.70 (s, 1H), 7.28 (t, *J* = 4.8 Hz, 1H), 7.18 (m, 2H), 6.96 (t, *J* = 7.5 Hz, 1H), 6.82 (d, *J* = 8.1 Hz, 1H), 6.74 (d, *J* = 2.3 Hz, 1H), 4.62 (s, 2H), 3.82 (s, 3H), 2.35 (s, 3H).

^{13}C NMR (151 MHz, $CDCl_3$) δ 166.13, 155.43, 145.90, 131.32, 131.18, 127.32, 126.90, 122.21, 111.82, 97.58, 67.73, 38.98, 16.61.

LCMS (Basic method) Rt 1.60 mins, MS m/z 246.2 [M+H]+

25 2-(2-Methylphenoxy)-*N*-(3-pyridylmethyl)acetamide

Prepared by procedure A with 2-methylphenoxyacetic acid (100 mg, 0.60 mmol) and 3-picolylamine (0.06 mL, 0.61 mmol). Product was isolated as white solid (120 mg, 78 %).

^{1}H NMR (600 MHz, $CDCl_3$) δ 8.60 – 8.49 (m, 2H), 7.63 (d, *J* = 7.9 Hz, 1H), 7.29 – 7.26 (m, 1H), 7.17 (t, *J* = 7.9 Hz, 2H), 6.94 (t, *J* = 7.3 Hz, 2H), 6.78 (d, *J* = 7.9 Hz, 1H), 4.61 – 4.54 (m, 4H), 2.24 (s, 3H).

^{13}C NMR (151 MHz, $CDCl_3$) δ 168.84, 155.37, 149.28, 149.19, 135.49, 133.57, 131.30, 127.37, 126.60, 123.77, 122.18, 111.69, 67.68, 40.59, 16.50.

LCMS (Basic method) Rt 1.56 mins, MS m/z 257.2 [M+H]+

26 2-(2-Methylphenoxy)-*N*-(indol-6-yl)acetamide

Prepared by procedure A with 2-methylphenoxyacetic acid (75 mg, 0.45 mmol) and 6-aminoindole (60 mg, 0.46 mmol). Product was isolated as a pale brown solid (82 mg, 65 %).

^{1}H NMR (600 MHz, $CDCl_3$) δ 8.44 (s, 1H), 7.60 (d, J = 7.8 Hz, 2H), 7.38 (t, J = 7.8 Hz, 2H), 7.25 – 7.19 (m, 2H), 7.17 (t, J = 7.4 Hz, 1H), 6.99 (t, J = 7.4 Hz, 1H), 6.86 (d, J = 8.0 Hz, 1H), 4.65 (s, 2H), 2.40 (s, 3H).

^{13}C NMR (151 MHz, $CDCl_3$) δ 166.33, 155.43, 136.04, 131.79, 131.33, 127.47, 126.65, 125.32, 124.76, 122.26, 121.02, 113.28, 112.00, 103.17, 102.67, 67.92, 16.56.

LCMS (Basic method) Rt 1.79 mins, MS m/z 242.1 [M+H]+

27 2-(2-Methylphenoxy)-*N*-(quinoxalin-6-yl)acetamide

Prepared by procedure A with 2-methylphenoxyacetic acid (75 mg, 0.45 mmol) and 6-aminoquinoxaline (67.00 mg, 0.46 mmol). Product isolated as an off-white solid (83 mg, 63 %).

^{1}H NMR (400 MHz, $CDCl_3$) δ 8.84 (d, *J* = 1.8 Hz, 1H), 8.79 (d, *J* = 1.8 Hz, 1H), 8.70 (s, 1H), 8.40 (d, *J* = 2.3 Hz, 1H), 8.11 (d, *J* = 9.1 Hz, 1H), 8.04 (dd, *J* = 9.1, 2.3 Hz, 1H), 7.25 – 7.19 (m, 2H), 7.00 (td, *J* = 7.5, 0.8 Hz, 1H), 6.87 (d, *J* = 8.1 Hz, 1H), 4.70 (s, 2H), 2.42 (s, 3H).

13C NMR (176 MHz, CDCl3) δ 166.94, 155.21, 145.80, 144.25, 143.76, 140.67, 138.33, 131.48, 130.61, 127.58, 126.61, 123.87, 122.58, 117.54, 112.04, 67.89, 16.59.

LCMS (Basic method) Rt 1.67 mins, MS m/z 294.1 [M+H]+

28 *N*-(1*H*-Indol-5-yl)-2-(2-methylphenoxy)acetamide

Prepared by procedure A with 2-methylphenoxyacetic acid (100 mg, 0.60 mmol) and 5-aminoindole (81 mg, 0.61 mmol). Product was isolated as a pale brown solid (109 mg, 65 %).

^{1}H NMR (600 MHz, $CDCl_3$) δ 8.37 (s, 1H), 8.22 (s, 1H), 7.92 (d, *J* = 2.0 Hz, 1H), 7.36 (d, *J* = 8.6 Hz, 1H), 7.30 (dd, *J* = 8.6, 2.0 Hz, 1H), 7.25 – 7.18 (m, 3H), 6.98 (td, *J* = 7.5, 0.7 Hz, 1H), 6.88 (d, *J* = 8.0 Hz, 1H), 6.55 (ddd, *J* = 3.0, 2.0, 0.7 Hz, 1H), 4.65 (s, 2H), 2.39 (s, 3H).

^{13}C NMR (151 MHz, $CDCl_3$) δ 166.43, 155.51, 133.60, 131.30, 129.62, 128.23, 127.48, 126.66, 125.44, 122.20, 116.30, 112.81, 112.00, 111.40, 103.05, 67.97, 16.57.

LCMS (Basic method) Rt 1.74 mins, MS m/z 281.2 [M+H]+

29 *N*-(1-Methylindol-5-yl)-2-(2-methylphenoxy)acetamide

Prepared by procedure A with 2-methylphenoxyacetic acid (75 mg, 0.45 mmol) and 5-amino-1-methyl-1*H*-indole (67 mg, 0.46 mmol). Product was isolated as a light brown solid (109 mg, 82 %).

^{1}H NMR (600 MHz, $CDCl_3$) δ 8.36 (s, 1H), 7.89 (d, *J* = 1.8 Hz, 1H), 7.34 (dd, *J* = 8.7, 1.8 Hz, 1H), 7.29 (d, *J* = 8.7 Hz, 1H), 7.24 – 7.19 (m, 2H), 7.07 (d, *J* = 3.0 Hz, 1H), 6.98 (t, *J* = 7.4 Hz, 1H), 6.88 (d, *J* = 8.0 Hz, 1H), 6.47 (d, *J* = 3.0 Hz, 1H), 4.65 (s, 2H), 3.79 (s, 3H), 2.40 (s, 3H).

^{13}C NMR (151 MHz, $CDCl_3$) δ 166.33, 155.52, 134.58, 131.28, 129.98, 129.27, 128.68, 127.46, 126.65, 122.16, 115.76, 112.86, 111.99, 109.55, 101.22, 67.97, 33.09, 16.57.

LCMS (Basic method) Rt 1.81 mins, MS m/z 295.1 [M+H]+

30 ***N*-(1-Methyl-1*H*-benzimidazol-5-yl)-2-(2-methylphenoxy)acetamide**

Prepared by procedure A with 2-methylphenoxyacetic acid (75 mg, 0.45 mmol) and 5-amino-1-methyl-1*H*-benzimdazole (69 mg, 0.47 mmol). Product was isolated as an off-white solid (101 mg, 76 %).

^{1}H NMR (300 MHz, $CDCl_3$) δ 8.45 (s, 1H), 7.94 (d, *J* = 2.0 Hz, 1H), 7.87 (s, 1H), 7.64 (dd, *J* = 8.7, 2.0 Hz, 1H), 7.37 (d, *J* = 8.7 Hz, 1H), 7.21 (t, *J* = 7.8 Hz, 2H), 7.02 – 6.93 (m, 1H), 6.87 (d, *J* = 7.8 Hz, 1H), 4.65 (s, 2H), 3.85 (s, 3H), 2.39 (s, 3H).

^{13}C NMR (176 MHz, $CDCl_3$) δ 166.53, 155.45, 144.65, 144.13, 132.32, 132.02, 131.36, 127.49, 126.68, 122.30, 117.08, 112.17, 112.03, 109.67, 67.95, 31.31, 16.57.

LCMS (Basic method) Rt 1.62 mins, MS m/z 296.1 [M+H]+

31 ***N*-(1-Methyl-1H-benzimidazol-6-yl)-2-(2-methylphenoxy)acetamide**

Prepared by procedure A with 2-methylphenoxyacetic acid (75 mg, 0.45 mmol) and 6-amino-1-methyl-1*H*-benzimdazole (69 mg, 0.47 mmol). Product was isolated as an off-white solid (97 mg, 73 %).

^{1}H NMR (600 MHz, $CDCl_3$) δ 8.51 (s, 1H), 8.24 (d, *J* = 1.9 Hz, 1H), 7.85 (s, 1H), 7.76 – 7.71 (m, 1H), 7.21 (ddd, *J* = 7.5, 4.8, 0.9 Hz, 2H), 7.03 – 6.95 (m, 2H), 6.87 (d, *J* = 8.0 Hz, 1H), 4.65 (s, 2H), 3.85 (s, 3H), 2.40 (s, 3H).

^{13}C NMR (151 MHz, $CDCl_3$) δ 166.52, 155.38, 144.20, 141.12, 135.02, 132.71, 131.40, 127.50, 126.66, 122.40, 120.64, 115.21, 112.04, 101.49, 67.94, 31.32, 16.55.

LCMS (Basic method) Rt 1.63 mins, MS m/z 296.1 [M+H]+

32 ***N*-(2-Methylindazol-5-yl)-2-(2-methylphenoxy)acetamide**

Prepared by procedure A with 2-methylphenoxyacetic acid (75 mg, 0.45 mmol) and 5-amino-2-methyl-2*H*-indazole (69 mg, 0.47 mmol). Product was isolated as a white solid (96 mg, 72 %).

^{1}H NMR (600 MHz, $CDCl_3$) δ 8.36 (s, 1H), 8.24 (d, *J* = 2.0 Hz, 1H), 7.87 (s, 1H), 7.67 (dt, *J* = 9.1, 0.8 Hz, 1H), 7.25 – 7.17 (m, 2H), 7.12 (dd, *J* = 9.1, 2.0 Hz, 1H), 6.98 (td, *J* = 7.4, 0.8 Hz, 1H), 6.87 (d, *J* = 8.0 Hz, 1H), 4.64 (s, 2H), 4.21 (s, 3H), 2.39 (s, 3H).

^{13}C NMR (151 MHz, $CDCl_3$) δ 166.51, 155.41, 131.36, 130.70, 127.50, 124.00, 122.33, 122.08, 121.35, 118.35, 112.03, 109.92, 67.93, 40.53, 16.55.

LCMS (Basic method) Rt 1.67 mins, MS m/z 296.2 [M+H]+

33 ***N*-(1-Methylindazol-5-yl)-2-(2-methylphenoxy)acetamide**

Prepared by procedure A with 2-methylphenoxyacetic acid (75 mg, 0.45 mmol) and 5-amino-1-methyl-1*H*-indazole (68 mg, 0.46 mmol). Product was isolated as an off-white solid (100 mg, 75 %).

^{1}H NMR (600 MHz, $CDCl_3$) δ 8.41 (s, 1H), 8.09 (d, *J* = 1.9 Hz, 1H), 7.96 (d, *J* = 0.6 Hz, 1H), 7.46 (dd, *J* = 8.9, 1.9 Hz, 1H), 7.38 (d, *J* = 8.9 Hz, 1H), 7.26 – 7.19 (m, 2H), 6.99 (t, *J* = 7.4 Hz, 1H), 6.88 (d, *J* = 8.0 Hz, 1H), 4.66 (s, 2H), 4.08 (s, 3H), 2.40 (s, 3H).

^{13}C NMR (151 MHz, $CDCl_3$) δ 166.62, 155.40, 137.79, 132.89, 131.35, 130.16, 127.50, 126.63, 124.16, 122.34, 120.96, 112.18, 112.03, 109.50, 67.94, 35.79, 16.57.

LCMS (Basic method) Rt 1.70 mins, MS m/z 296.1 [M+H]+

34 *N*-(1-Methylindazol-6-yl)-2-(2-methylphenoxy)acetamide

Prepared by procedure A with 2-methylphenoxyacetic acid (100 mg, 0.60 mmol) and 1-methylindazol-6-amine (90 mg, 0.61 mmol). Product was isolated as a white solid (141 mg, 79 %).

^{1}H NMR (700 MHz, CDCl3) δ 8.53 (s, 1H), 8.22 (s, 1H), 7.93 (d, J = 0.9 Hz, 1H), 7.66 (dd, J = 8.5, 0.5 Hz, 1H), 7.26 – 7.19 (m, 2H), 6.99 (td, J = 7.4, 0.5 Hz, 1H), 6.91 – 6.85 (m, 2H), 4.66 (s, 2H), 4.08 (s, 3H), 2.40 (s, 3H).

^{13}C NMR (176 MHz, CDCl3) δ 166.76, 155.33, 140.47, 135.38, 132.79, 131.42, 127.55, 126.64, 122.48, 121.77, 121.33, 114.38, 112.08, 99.65, 67.96, 35.80, 16.58.

LCMS (Basic method) Rt 1.75 mins, MS m/z 296.2 [M+H]+

HRMS (m/z TOF MS ES^+) for $C_{17}H_{17}N_3O_2$ $[M+H]^+$ calc. 296.1394, observed 296.1393.

35 *N*-(2-Methylindazol-6-yl)-2-(2-methylphenoxy)acetamide

Prepared by procedure A with 2-methylphenoxyacetic acid (75 mg, 0.45 mmol) and 2-methylindazol-6-amine (67 mg, 0.46 mmol). Product was then further purified by normal phase chromatography (10-100 %, EtOAc:cyclohexane) and isolated as an off-white solid (95 mg, 71 %).

^{1}H NMR (600 MHz, $CDCl_3$) δ 8.42 (s, 1H), 8.07 (s, 1H), 7.86 (s, 1H), 7.63 (d, *J* = 8.9 Hz, 1H), 7.25 – 7.16 (m, 3H), 6.98 (t, *J* = 7.4 Hz, 1H), 6.87 (d, *J* = 8.1 Hz, 1H), 4.65 (s, 2H), 4.21 (s, 3H), 2.39 (s, 3H).

^{13}C NMR (151 MHz, $CDCl_3$) δ 166.52, 155.38, 149.21, 134.74, 131.33, 127.48, 126.63, 123.81, 122.30, 120.94, 119.87, 117.10, 112.03, 106.76, 67.91, 40.48, 16.53.

LCMS (Basic method) Rt 1.65 mins, MS m/z 294.1 [M+H]+

36 *N*-(1-Ethylindazol-6-yl)-2-(2-methylphenoxy)acetamide

Prepared by procedure A with 2-methylphenoxyacetic acid (70 mg, 0.42 mmol) and 1-ethylindazol-6-amine (69 mg, 0.43 mmol). Product isolated as an off-white solid (110 mg, 84 %).

^{1}H NMR (600 MHz, CDCl3) δ 8.52 (s, 1H), 8.25 (s, 1H), 7.94 (s, 1H), 7.66 (d, J= 8.5 Hz, 1H), 7.24 (d, J = 7.4 Hz, 1H), 7.22 (d, J = 7.4 Hz, 1H), 6.99 (t, J= 7.4 Hz, 1H), 6.88 (d, J= 8.5 Hz, 2H), 4.66 (s, 2H), 4.44 (q, J= 7.3 Hz, 2H), 2.40 (s, 3H), 1.52 (t, J= 7.3 Hz, 3H).

^{13}C NMR (151 MHz, CDCl3) δ 166.71, 155.31, 139.51, 135.23, 132.83, 131.40, 127.52, 126.63, 122.45, 121.79, 121.39, 114.31, 112.07, 99.61, 67.95, 43.85, 16.56, 15.01.

LCMS (Basic method) Rt 1.78 mins, MS m/z 310.2 [M+H]+

37*N*-(2-Ethylindazol-6-yl)-2-(2-methylphenoxy)acetamide

Prepared by procedure A with 2-methylphenoxyacetic acid (75 mg, 0.45 mmol) and 2-ethylindazol-6-amine (74 mg, 0.46 mmol). Product isolated as an off-white solid (102 mg, 73 %).

^{1}H NMR (600 MHz, $CDCl_3$) δ 8.42 (s, 1H), 8.08 (s, 1H), 7.90 (s, 1H), 7.63 (d, *J* = 8.9 Hz, 1H), 7.26 – 7.13 (m, 3H), 6.98 (t, *J* = 7.4 Hz, 1H), 6.87 (d, *J* = 8.1 Hz, 1H), 4.65 (s, 2H), 4.46 (q, *J* = 7.4 Hz, 2H), 2.39 (s, 3H), 1.64 (t, *J* = 7.4 Hz, 3H).

^{13}C NMR (151 MHz, $CDCl_3$) δ 166.47, 155.38, 148.95, 134.68, 131.33, 127.48, 126.61, 122.28, 122.12, 121.04, 119.59, 116.98, 112.00, 106.89, 67.89, 48.66, 16.52, 15.93.

LCMS (Basic method) Rt 1.70 mins, MS m/z 308.2 [M+H]+

38 *N*-(2-Isopropylindazol-6-yl)-2-(2-methylphenoxy)acetamide

Prepared by procedure A with 2-methylphenoxyacetic acid (70 mg, 0.42 mmol) and 2-isopropylindazol-6-amine (74 mg, 0.42 mmol). Isolated as an off-white solid (93 mg, 68 %).

^{1}H NMR (600 MHz, $CDCl_3$) δ 8.42 (s, 1H), 8.07 (s, 1H), 7.92 (s, 1H), 7.63 (d, *J* = 8.9 Hz, 1H), 7.21 (m, 3H), 6.98 (t, *J* = 7.4 Hz, 1H), 6.86 (d, *J* = 8.1 Hz, 1H), 4.77 (hept, *J* = 6.7 Hz, 1H), 4.64 (s, 2H), 2.39 (s, 3H), 1.65 (d, *J* = 6.7 Hz, 6H).

^{13}C NMR (151 MHz, $CDCl_3$) δ 166.42, 155.37, 148.52, 134.62, 131.32, 127.48, 126.59, 122.26, 121.10, 120.26, 119.27, 116.84, 111.96, 106.95, 67.85, 55.55, 23.37, 16.52.

LCMS (Basic method) Rt 1.70 mins, MS m/z 324.2 [M+H]+

HRMS (m/z TOF MS ES$^+$) for $C_{19}H_{21}N_3O_2$ $[M+H]^+$ calc. 324.1707, observed 324.1704.

39 *N*-(3*H*-Benzotriazol-5-yl)-2-(2-methylphenoxy)acetamide

Prepared by procedure A with 2-methylphenoxyacetic acid (75 mg, 0.45 mmol) and 1*H*-1,2,3-benzotriazol-5-amine (62 mg, 0.46 mmol). Isolated as a white solid (61 mg, 48 %).

^{1}H NMR (600 MHz, MeOD) δ 8.40 (d, *J* = 1.1 Hz, 1H), 7.87 (d, *J* = 8.9 Hz, 1H), 7.48 (d, *J* = 8.9 Hz, 1H), 7.22 – 7.11 (m, 2H), 6.91 (t, *J* = 7.4 Hz, 2H), 4.73 (s, 2H), 2.35 (s, 3H).

^{13}C NMR (151 MHz, MeOD) δ 169.94, 157.52, 131.98, 128.25, 128.05, 122.77, 112.90, 69.15, 16.42.

A number of quaternary carbons were not seen, however the ^{1}H NMR and LCMS confirmed the product as clean and correct.

LCMS (Basic method) Rt 1.35 mins, MS m/z 283.1 [M+H]+

HRMS (m/z TOF MS ES$^+$) for $C_{15}H_{14}N_4O_2$ $[M+H]^+$ calc. 283.1190, observed 283.1189.

40 *N*-(3-Methylbenzotriazol-5-yl)-2-(2-methylphenoxy)acetamide

Prepared by procedure A with 2-methylphenoxyacetic acid (75 mg, 0.45 mmol) and 3-methylbenzotriazol-5-amine (70 mg, 0.47 mmol). Product was isolated as an off-white solid (13 mg, 10 %).

^{1}H NMR (700 MHz, $CDCl_3$) δ 8.63 (s, 1H), 8.44 (s, 1H), 7.99 (d, *J* = 8.7 Hz, 1H), 7.23 (m, 2H), 7.05 (d, *J* = 8.7 Hz, 1H), 7.00 (t, *J* = 7.7 Hz, 1H), 6.87 (d, *J* = 8.1 Hz, 1H), 4.67 (s, 2H), 4.30 (s, 3H), 2.41 (s, 3H).

^{13}C NMR (176 MHz, $CDCl_3$) δ 166.74, 155.32, 144.98, 142.33, 134.96, 131.42, 127.55, 126.64, 122.48, 121.56, 118.75, 112.10, 107.26, 67.94, 43.38, 16.57.

LCMS (Basic method) Rt 1.70 mins, MS m/z 297.1 [M+H]+

41 *N*-(2-Methylbenzotriazol-5-yl)-2-(2-methylphenoxy)acetamide

Prepared by procedure A with 2-methylphenoxyacetic acid (75 mg, 0.45 mmol) and 2-methylbenzotriazol-5-amine (70 mg, 0.47 mmol). Product was isolated as an off-white solid (87 mg, 65 %).

^{1}H NMR (700 MHz, $CDCl_3$) δ 8.49 (s, 1H), 8.37 (s, 1H), 7.83 (d, *J* = 9.0 Hz, 1H), 7.35 (d, *J* = 9.0 Hz, 1H), 7.22 (m, 2H), 6.99 (t, *J* = 7.4 Hz, 1H), 6.87 (d, *J* = 8.1 Hz, 1H), 4.66 (s, 2H), 4.50 (s, 3H), 2.40 (s, 3H).

^{13}C NMR (176 MHz, $CDCl_3$) δ 166.74, 155.32, 144.98, 142.33, 134.96, 131.42, 127.55, 126.64, 122.48, 121.56, 118.75, 112.10, 107.26, 67.94, 43.38, 16.57.

LCMS (Basic method) Rt 1.73 mins, MS m/z 297.1 [M+H]+

42 *N*-(3-Isoquinolyl)-2-(2-methylphenoxy)acetamide

Prepared by procedure A with 2-methylphenoxyacetic acid (100 mg, 0.60 mmol) and 3-aminoisoquinoline (87 mg, 0.61 mmol). Product was isolated as a yellow solid (84 mg, 48 %).

^{1}H NMR (700 MHz, DMSO) δ 10.50 (s, 1H), 9.16 (s, 1H), 8.47 (s, 1H), 8.06 (dd, *J* = 8.2, 1H), 7.90 (d, *J* = 8.2 Hz, 1H), 7.71 (ddd, *J* = 8.2, 6.8, 1.2 Hz, 1H), 7.54 (ddd, *J* = 8.2, 6.8, 1.2 Hz, 1H), 7.20 – 7.09 (m, 2H), 6.95 – 6.82 (m, 2H), 4.86 (s, 2H), 2.26 (s, 3H).

^{13}C NMR (176 MHz, DMSO) δ 167.10, 155.92, 151.47, 146.38, 137.10, 131.00, 130.60, 127.60, 126.91, 126.38, 125.99, 125.96, 125.88, 120.84, 111.39, 106.97, 66.88, 16.09.

LCMS (Basic method) Rt 1.92 mins, MS m/z 293.1 [M+H]+

43 2-(2-Methylphenoxy)-*N*-(3-quinolyl)acetamide

Prepared by procedure A with 2-methylphenoxyacetic acid (75 mg, 0.45 mmol) and 3-aminoquinoline (66 mg, 0.46 mmol). Product was isolated as an off-white solid (97 mg, 74 %).

^{1}H NMR (700 MHz, $CDCl_3$) δ 8.82 (d, *J* = 2.2 Hz, 1H), 8.79 (d, *J* = 2.2 Hz, 1H), 8.61 (s, 1H), 8.07 (d, *J* = 8.3 Hz, 1H), 7.85 (d, *J* = 8.3 Hz, 1H), 7.67 (t, *J* = 7.4 Hz, 1H), 7.57 (t, *J* = 7.4 Hz, 1H), 7.26 – 7.16 (m, 2H), 7.01 (t, *J* = 7.4 Hz, 1H), 6.89 (d, *J* = 8.3 Hz, 1H), 4.71 (s, 2H), 2.42 (s, 3H).

^{13}C NMR (176 MHz, $CDCl_3$) δ 167.32, 155.28, 145.76, 144.00, 131.52, 130.50, 129.33, 128.72, 128.22, 127.94, 127.57, 126.74, 124.29, 122.66, 112.19, 68.04, 16.60.

LCMS (Basic method) Rt 1.74 mins, MS m/z 293.2 [M+H]+

44 2-(2-Methylphenoxy)-*N*-(6-quinolyl)acetamide

Prepared by procedure A with 2-methylphenoxyacetic acid (75 mg, 0.45 mmol) and 6-aminoquinoline (66 mg, 0.46 mmol). Product was further purified by normal phase chromatography (10-100 %, EtOAc:Cyclohexane) and isolated as a white solid (52 mg, 39 %).

^{1}H NMR (600 MHz, $CDCl_3$) δ 8.87 (dd, *J* = 4.2, 1.6 Hz, 1H), 8.58 (s, 1H), 8.43 (d, *J* = 2.4 Hz, 1H), 8.17 (d, *J* = 7.9 Hz, 1H), 8.10 (d, *J* = 9.0 Hz, 1H), 7.63 (dd, *J* = 9.0, 2.4 Hz, 1H), 7.41 (dd, *J* = 8.3, 4.2 Hz, 1H), 7.23 (m, 2H), 7.00 (t, *J* = 7.4 Hz, 1H), 6.88 (d, *J* = 7.9 Hz, 1H), 4.69 (s, 2H), 2.42 (s, 3H).

^{13}C NMR (151 MHz, $CDCl_3$) δ 166.86, 155.28, 149.86, 145.94, 136.00, 134.82, 131.43, 130.64, 128.91, 127.54, 126.62, 123.19, 122.50, 121.90, 116.48, 112.06, 67.94, 16.58.

LCMS (Basic method) Rt 1.70 mins, MS m/z 293.1 [M+H]+

45 *N*-(6-Isoquinolyl)-2-(2-methylphenoxy)acetamide

Prepared by procedure A with 2-methylphenoxyacetic acid (75 mg, 0.45 mmol) and 6-aminoisoquinoline (66 mg, 0.46 mmol). Product was further purified by normal phase chromatography (15-100 %, EtOAc:CH_2Cl_2) and isolated as a white solid (47 mg, 36 %).

^{1}H NMR (600 MHz, CDCl3) δ 9.18 (s, 1H), 8.62 (s, 1H), 8.51 (d, J = 5.8 Hz, 1H), 8.35 (d, J = 1.8 Hz, 1H), 7.97 (d, J = 8.8 Hz, 1H), 7.64 (d, J = 5.8 Hz, 1H), 7.59 (dd, J = 8.8, 1.8 Hz, 1H), 7.23 (m, 2H), 7.00 (t, J = 7.4 Hz, 1H), 6.88 (d, J = 8.1 Hz, 1H), 4.68 (s, 2H), 2.42 (s, 3H).

^{13}C NMR (176 MHz, CDCl3) δ 167.05, 155.26, 152.06, 143.94, 138.27, 136.90, 131.48, 129.17, 127.60, 126.64, 126.20, 122.62, 120.94, 120.53, 114.77, 112.14, 68.00, 16.60.

LCMS (Basic method) Rt 1.72 mins, MS m/z 293.2 [M+H]+

HRMS (m/z TOF MS ES^+) for $C_{18}H_{16}N_2O_2$ $[M+H]^+$ calc. 293.1285, observed 293.1283.

46 *N*-(7-Isoquinolyl)-2-(2-methylphenoxy)acetamide

Prepared by procedure A with 2-methylphenoxyacetic acid (100 mg, 0.60 mmol) and 7-aminoisoquinoline (87 mg, 0.61 mmol). Product was isolated as a yellow solid (70 mg, 40 %).

^{1}H NMR (700 MHz, DMSO-d_6) δ 10.44 (s, 1H), 9.23 (s, 1H), 8.52 (d, *J* = 1.9 Hz, 1H), 8.41 (d, *J* = 5.6 Hz, 1H), 7.94 (d, *J* = 8.9 Hz, 1H), 7.86 (dd, *J* = 8.9, 1.9 Hz, 1H), 7.75 (d, *J* = 5.6 Hz, 1H), 7.20 – 7.11 (m, 2H), 6.93 – 6.84 (m, 2H), 4.80 (s, 2H), 2.27 (s, 3H).

^{13}C NMR (176 MHz, DMSO-d_6) δ 167.26, 156.02, 151.80, 141.83, 137.13, 131.97, 130.61, 128.66, 127.28, 126.90, 126.17, 124.55, 120.93, 119.96, 114.95, 111.50, 67.40, 16.14.

LCMS (Basic method) Rt 1.73 mins, MS m/z 293.1 [M+H]+

47 2-(2-Methylphenoxy)-*N*-(7-quinolyl)acetamide

Prepared by procedure A with 2-methylphenoxyacetic acid (75 mg, 0.45 mmol) and 7-aminoquinoline (66 mg, 0.46 mmol). Product was isolated as a white solid (76 mg, 58 %).

^{1}H NMR (600 MHz, $CDCl_3$) δ 8.91 (dd, *J* = 4.2, 2.0 Hz, 1H), 8.66 (s, 1H), 8.19 (d, *J* = 2.0 Hz, 1H), 8.13 (d, *J* = 8.1 Hz, 1H), 8.02 (dd, *J* = 8.8, 2.0 Hz, 1H), 7.84 (d, *J* = 8.8 Hz, 1H), 7.36 (dd, *J* = 8.1, 4.2 Hz, 1H), 7.23 (m, 2H), 7.00 (t, *J* = 7.4 Hz, 1H), 6.88 (d, *J* = 8.1 Hz, 1H), 4.69 (s, 2H), 2.42 (s, 3H).

^{13}C NMR (151 MHz, $CDCl_3$) δ 166.78, 155.21, 151.36, 148.81, 138.07, 135.86, 131.42, 129.01, 127.52, 126.58, 125.78, 122.42, 120.56, 120.51, 117.82, 111.87, 67.75, 16.60.

LCMS (Basic method) Rt 1.72 mins, MS m/z 293.1 [M+H]+

48 2-(2-Methylphenoxy)-*N*-(2-quinolyl)acetamide

Prepared by procedure A with 2-methylphenoxyacetic acid (100 mg, 0.60 mmol) and 2-aminoquinoline (87 mg, 0.61 mmol). Product was isolated as a yellow solid (130 mg, 74 %).

^{1}H NMR (700 MHz, DMSO-d_6) δ 10.84 (s, 1H), 8.37 (d, *J* = 8.9 Hz, 1H), 8.25 (d, *J* = 7.6 Hz, 1H), 7.91 (dd, *J* = 8.1, 1.1 Hz, 1H), 7.82 (dd, *J* = 8.1, 1.1 Hz, 1H), 7.71 (ddd, *J* = 8.1, 6.9, 1.1 Hz, 1H), 7.50 (ddd, *J* = 8.1, 6.9, 1.1 Hz, 1H), 7.21 – 7.10 (m, 2H), 6.91 – 6.81 (m, 2H), 4.89 (s, 2H), 2.23 (s, 3H).

^{13}C NMR (176 MHz, DMSO-d_6) δ 168.02, 155.97, 150.96, 146.26, 138.53, 130.60, 130.09, 127.77, 126.99, 126.89, 125.97, 125.72, 125.09, 120.81, 114.12, 111.32, 66.88, 16.09.

LCMS (Basic method) Rt 1.92 mins, MS m/z 293.1 [M+H]+

49 2-(2-Methylphenoxy)-*N*-(7-quinazolinyl)acetamide

Prepared by procedure A with 2-methylphenoxyacetic acid (70 mg, 0.42 mmol) and quinazolin-7-amine (62 mg, 0.43 mmol). Product was isolated as a white solid (65 mg, 53 %).

^{1}H NMR (600 MHz, $CDCl_3$) δ 9.32 (s, 1H), 9.30 (s, 1H), 8.75 (s, 1H), 8.24 (d, *J* = 2.0 Hz, 1H), 8.03 (dd, *J* = 8.8, 2.0 Hz, 1H), 7.95 (d, *J* = 8.8 Hz, 1H), 7.26 – 7.20 (m, 2H), 7.00 (t, *J* = 7.4 Hz, 1H), 6.87 (d, *J* = 8.1 Hz, 1H), 4.70 (s, 2H), 2.42 (s, 3H).

^{13}C NMR (151 MHz, $CDCl_3$) δ 167.30, 159.70, 156.41, 155.35, 151.40, 142.33, 131.74, 128.97, 127.82, 126.82, 122.90, 122.76, 121.79, 116.23, 112.26, 68.09, 16.81.

LCMS (Basic method) Rt 1.66 mins, MS m/z 294.1 [M+H]+

50 2-(2-Chlorophenoxy)-*N*-(6-isoquinolyl)acetamide

Prepared by procedure A with 2-chlorophenoxyacetic acid (100 mg, 0.54 mmol) and 6-aminoisoquinoline (77 mg, 0.54 mmol). Product was isolated as an off-white solid (113 mg, 67 %).

^{1}H NMR (600 MHz, DMSO-d_6) δ 10.59 (s, 1H), 9.19 (s, 1H), 8.43 (d, *J* = 5.7 Hz, 1H), 8.38 (d, *J* = 1.3 Hz, 1H), 8.09 (d, *J* = 8.9 Hz, 1H), 7.78 – 7.69 (m, 2H), 7.47 (dd, *J* = 7.9, 1.3 Hz, 1H), 7.37 – 7.25 (m, 1H), 7.13 (dd, *J* = 8.3, 1.3 Hz, 1H), 7.00 (td, *J* = 7.9, 1.3 Hz, 1H), 4.94 (s, 2H).

^{13}C NMR (151 MHz, DMSO-d_6) δ 166.81, 153.46, 151.65, 143.39, 139.86, 136.15, 130.15, 128.74, 128.31, 125.21, 122.19, 121.48, 121.16, 120.14, 114.12, 113.38, 67.62.

LCMS (Basic method) Rt 1.74 mins, MS m/z 313.1 [M+H]+

HRMS (m/z TOF MS ES$^+$) for $C_{17}H_{13}N_2O_2Cl$ $[M+H]^+$ calc. 313.0738, observed 313.0737.

51 *N*-(6-Isoquinolyl)-2-(3-methylphenoxy)acetamide

Prepared by procedure A with 3-methylphenoxyacetic acid (100 mg, 0.60 mmol) and 6-aminoisoquinoline (87 mg, 0.60 mmol). Product was isolated as a yellow solid (131 mg, 75 %).

^{1}H NMR (600 MHz, DMSO-d_6) δ 10.48 (s, 1H), 9.19 (s, 1H), 8.42 (d, *J* = 5.7 Hz, 1H), 8.39 (d, *J* = 1.3 Hz, 1H), 8.08 (d, *J* = 8.9 Hz, 1H), 7.80 (dd, *J* = 8.9, 2.0 Hz, 1H), 7.74 (d, *J* = 5.7 Hz, 1H), 7.20 (t, *J* = 7.9 Hz, 1H), 6.86 (br s, 1H), 6.84 – 6.78 (m, 2H), 4.77 (s, 2H), 2.29 (s, 3H).

^{13}C NMR (151 MHz, DMSO-d_6) δ 167.49, 157.85, 151.64, 143.35, 139.90, 139.08, 136.10, 129.31, 128.61, 125.23, 122.04, 121.43, 120.14, 115.49, 113.62, 111.61, 67.14, 21.15.

LCMS (Acidic method) Rt 1.39 mins, MS m/z 293.1 [M+H]+

52 2-(3-Chlorophenoxy)-*N*-(6-isoquinolyl)acetamide

Prepared by procedure A with 3-chlorophenoxyacetic acid (100 mg, 0.54 mmol) and 6-aminoisoquinoline (77 mg, 0.54 mmol). Product was isolated as an off-white solid (115 mg, 69 %).

^{1}H NMR (600 MHz, DMSO-d_6) δ 10.52 (s, 1H), 9.19 (s, 1H), 8.43 (d, *J* = 5.7 Hz, 1H), 8.39 (d, *J* = 1.2 Hz, 1H), 8.09 (d, *J* = 8.8 Hz, 1H), 7.78 (dd, *J* = 8.8, 2.0 Hz, 1H), 7.75 (d, *J* = 5.7 Hz, 1H), 7.35 (t, *J* = 8.2 Hz, 1H), 7.14 (t, *J* = 2.0 Hz, 1H), 7.05 (dd, *J* = 8.2, 1.2 Hz, 1H), 7.02 (dd, *J* = 8.2, 2.0 Hz, 1H), 4.85 (s, 2H).

^{13}C NMR (151 MHz, DMSO-d_6) δ 167.01, 158.79, 151.65, 143.37, 139.82, 136.10, 133.70, 130.97, 128.66, 125.25, 121.38, 121.24, 120.15, 115.05, 113.73, 113.65, 67.24.

LCMS (Basic method) Rt 1.74 mins, MS m/z 313.1 [M+H]+

53 2-(*N*,2-dimethylanilino)-*N*-(1-methylindazol-6-yl)acetamide

In a 20 mL microwave vial, *N*,2-dimethylaniline (0.04 mL, 0.36 mmol), potassium carbonate (74 mg, 0.54 mmol) and 2-chloro-*N*-(1-methylindazol-6-yl)acetamide (80 mg, 0.36 mmol) were dissolved in *N,N*-dimethylformamide (2mL) and stirred for 30 minutes. To this solution potassium iodide (59 mg, 0.36 mmol) was added and the reaction was sealed and heated to 80 °C for 4 h. After this time, the reaction was cooled to RT and purified directly by reverse phase chromatography (15-100 %, MeCN:H_2O, 10 mM NH_4OH modifier) to give the product as a yellow oil (22 mg, 20 %).

^{1}H NMR (400 MHz, methanol-d_4) δ 8.16 – 8.02 (m, 1H), 7.93 (d, *J* = 0.7 Hz, 1H), 7.69 (d, *J* = 8.7 Hz, 1H), 7.19 (m, 3H), 7.13 (dd, *J* = 8.7, 1.7 Hz, 1H), 7.00 (td, *J* = 7.1, 1.7 Hz, 1H), 4.02 (s, 3H), 3.75 (s, 2H), 2.83 (s, 3H), 2.44 (s, 3H).

^{13}C NMR (176 MHz, methanol-d_4) δ 170.26, 151.13, 140.37, 136.82, 132.41, 132.34, 131.18, 126.63, 123.87, 121.30, 120.87, 119.96, 114.95, 99.37, 60.68, 42.04, 34.21, 17.40.

LCMS (Basic method) Rt 1.70 mins, MS m/z 309.1 [M+H]+

HRMS (m/z TOF MS ES$^+$) for $C_{18}H_{20}N_4O$ $[M+H]^+$ calc. 309.1710, observed 309.1710.

54 1-(3,4-Dihydro-1*H*-isoquinolin-2-yl)-2-(2-methylphenoxy)ethanone

Prepared by procedure A with 2-methylphenoxyacetic acid (75 mg, 0.45 mmol) and 1,2,3,4-tetrahydroisoquinoline (63 mg, 0.47 mmol). Product isolated as a light yellow gel (96 mg, 76 %).

^{1}H NMR (300 MHz, $CDCl_3$) δ 7.25 – 7.05 (m, 6H), 6.96 – 6.82 (m, 2H), 4.77 (m, 4H), 3.85 (m, 2H), 2.90 (m, 2H), 2.26 (seen as 2 rotameric singlets, 3H).

^{13}C NMR (176 MHz, $CDCl_3$) δ 167.44, 167.26, 156.17, 156.12, 134.92, 134.04, 132.89, 132.59, 131.10, 129.12, 128.57, 127.18, 127.16, 127.10, 126.97, 126.82, 126.78, 126.76, 126.73, 126.56, 126.17, 121.47, 111.26, 111.18, 68.40, 68.29, 47.17, 44.83, 43.21, 40.53, 29.77, 28.42, 16.48, 16.36. (Seen as rotameric).

LCMS (Basic method) Rt 1.85 mins, MS m/z 282.1 [M+H]+

HRMS (m/z TOF MS ES$^+$) for $C_{18}H_{19}NO_2$ $[M+H]^+$ calc. 282.1489, observed 282.1491.

NOTUM Protein Expression and Purification:

Nomenclature

NOTUM (EC 3.1.1.98) is also known as *O*-palmitoleoyl-L-serine hydrolase.

Preparation of NOTUM protein using HEK293S (GnTI$^-$) stably transfected with pNeo sec-hNotum (core)

These cells constitutively express NOTUM core protein (a full functional secreted protein spanning residues 81-451 of NOTUM with mutation Cys330Ser as previously described).[1] They are G418 resistant but G418 is not needed for culture.

The HEK293S cell line was sourced from ATCC: HEK293S GnTI$^-$ (ATCC® CRL-3022™).

Growth Media:

DMEM (High Glucose), 5mM Glutamine, 1x NEAA, 10 % FCS.

Production Media:

DMEM (High Glucose), 5mM Glutamine, 1x NEAA, 1.5 % - 2 % FCS.

Culture:

Culture cells in growth media up to required quantity at 37 °C. The cells can be expanded either using corrugated roller bottles, or triple layer flasks.

For Roller Bottles – Subculture from starter culture (in stages) up to approximately 12 xT175 flasks before expanding up to 25 x Corrugated roller bottles, adding 250 mL of complete growth medium to each roller bottle. After approximately 72 hours the medium should be changed to the Production Medium. Leave for 7-10 days before harvesting.

For Triple Layer Flasks – Subculture from starter culture (in stages) up to approximately 60 xT175 flasks before expanding up to 100 x triple layer flasks, adding 170 mL of complete growth medium to each roller bottle. After approximately 72 hours the medium should be changed to the Production Medium. Leave for 10 days before harvesting.

Harvesting:

Pour off the supernatant and spin at 2000 rpm to remove cell debris. Pass through 0.22 μM filter to get rid of aggregates. Use a 10K MWCO PES Membrane Filter (e.g. VivaFlow from Sartorius Cat: VF20P0) to concentrate the supernatant 10 fold. Re-filter through a 0.22 μM filter to ensure sterility. Keep at +4 °C short term.

Dialysis:

Using Thermo Fisher Slide-A-Lyzer Dialysis Flasks (e.g. 2K MWCO 250 mL from Fisher Cat: 87760) the concentrated supernatant now needs to be dialysed extensively against PBS overnight (15 litres of PBS with 300 mM NaCl for each litre of media). This should be repeated once. The dialysed supernatant requires centrifugation at 2000rpm for 15 minutes and re-filtering through a 0.22 μM filter to retain sterility. Keep at +4 °C short term.

Long-term storage:

Aliquot, add 10 % glycerol and store in -80 ^{o}C.

Protein purification:

NOTUM was purified from conditioned culture supernatants essentially as described.[1] In brief, the conditioned supernatant collected from a cell-line secreting 6xHis tagged NOTUM(81-451 Cys330Ser) was incubated with NiNTA resin (Qiagen) and incubated overnight at 4 °C gently rocking. Subsequently, the resin was washed 5 times with PBS before NOTUM was eluted with 250 mM imidazole. The eluate was concentrated and applied to a Superdex75 (GE Healthcare) and the NOTUM containing fractions were pooled, concentrated and snap-frozen in liquid N_2 and stored at -80 ^{o}C until used.

OPTS Biochemical NOTUM enzymatic activity assay

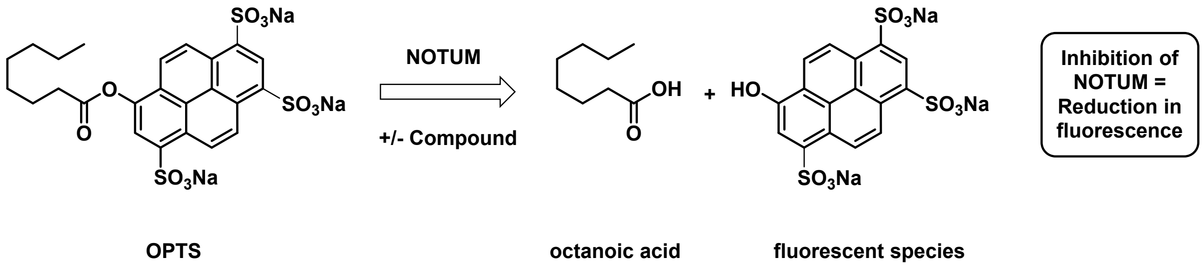

Figure S1: Chemical structures and representation of the OPTS biochemical NOTUM assay.

An Echo liquid handler was used to acoustically dispense 500 nL of compounds into dry Greiner 384-well plates (catalog #781076), followed by 25 µL of 2 µM trisodium 8-octanoyloxypyrene-1,3,6-trisulfonate (OPTS, Sigma #74875) solution in 50 mM Tris, 5 mM $CaCl_2$, 0.5 mM $MgCl_2$, pH 7.4 assay buffer, and 25 µL of 2.38 nM NOTUM carboxyesterase enzyme solution in the same buffer to every well in the assay plate. After 1 hour incubation at room temperature, fluorescence was measured on a PheraSTAR FSX microplate reader with an excitation wavelength of 485 nm and emission wavelength of 520 nm.

Dose-response curve analysis: Plate layout and measured fluorescence values were input into Dotmatics Studies data management tool. After implementing Quality Control with a cut-off of Z prime values higher than 0.5, compound IC_{50} values were estimated and compared between technical replicates. Final potency values implied an n ≥ 2 with IC_{50} within 0.5-2x between replicates.

Table S1. Inhibition of NOTUM activity.[a]

Compound #	NOTUM inhibition IC_{50} (µM)
**3**	33 ± 4.7
**4**	60 ± 10
**5**	110 ± 27
**6**	140 ± 7
**7**	23 ± 2.7
**8**	150 ± 1.4
**9**	290 ± 0.5
**10**	170 ± 4
**11**	120 ± 52
**12**	37 ± 1
**13**	14 ± 0.7
**14**	310 ± 14
**15**	ca. 25-50 % I @ 100 µM
**16**	ca. 20 % I @ 100 µM
**17**	20 ± 1.9
**18**	44 ± 2.3
**19**	180 ± 2.6
**20**	100 ± 1.7

**21**	1.6 ± 0.1
**22**	9.4 ± 1.2
**23**	3.6 ± 0.4
**24**	72 ± 7
**25**	100 ± 6.6
**26**	0.21 ± 0.06
**27**	0.68 ± 0.17
**28**	0.33 ± 0.05
**29**	0.24 ± 0.04
**30**	0.52 ± 0.09
**31**	0.98 ± 0.12
**32**	0.36 ± 0.01
**33**	0.27 ± 0.17
**34**	0.27 ± 0.06
**35**	0.28 ± 0.04
**36**	0.20 ± 0.01
**37**	0.068 ± 0.014
**38**	0.032 ± 0.004
**39**	0.12 ± 0.04
**40**	0.44 ± 0.01
**41**	0.27 ± 0.01
**42**	7.4 ± 1.3
**43**	2.5 ± 0.23
**44**	1.2 ± 0.25
**45**	0.085 ± 0.011
**46**	0.97 ± 0.12
**47**	0.43 ± 0.05
**48**	15 ± 1.7
**49**	0.67 ± 0.11
**50**	0.069 ± 0.011
**51**	19 ± 2
**52**	5.2 ± 0.6
**53**	5.7 ± 1.8
**54**	7.9 ± 2.1

[a] Values are geometric mean ± s.e.m of n = 2-8 experiments quoted to 2 s.f. Screening data only passed the quality control criteria if the screening plates demonstrated a Z' > 0.5 and the NOTUM inhibition activity of the positive control was within acceptable limits (IC_{50} 0.6 – 1.1 nM). Differences of <2-fold should not be considered significant.

FP-Biotin competition assay

Conditioned media was collected from HEK293S cells stably overexpressing human full length NOTUM. Cells were seeded in a T175 flask and grown to between 80-90 % confluency in DMEM (high glucose, GlutaMAX, supplement pyruvate) with 10 % fetal bovine serum and 10,000 u/mL penicillin/streptomycin. Media was then removed and the cells washed 3X with PBS and then incubated with serum-free DMEM for 48 hours. Media was then removed and cellular debris pelleted (300 g, 3 mins) and the supernatant snap frozen and stored at -80 °C. When ready for use media was defrosted at room temperature then applied to 10k MWCO filter (Thermo Fisher, 88527) and concentrated at 4 °C (4,000 RPM, 60 mins, Beckman JS 4.2 centrifuge). Samples were then diluted in PBS to an appropriate volume then aliquoted. These were then treated with either DMSO or inhibitor then equilibrated for 30 mins at room temperature. FP-biotin (2 µM, Santa Cruz) or DMSO was then added to the samples for 10 mins at room temperature. The reaction was halted by the addition of 4X LDS sample buffer, 10X reducing agent and then heating samples to 75 °C for 10 mins. Samples were then analysed by SDS-PAGE (Thermo Fisher, Bolt 4-12% Bis-Tris Plus gels), transferred onto nitrocellulose then samples blocked in 5% BSA TBST. Membranes were then incubated with Streptavidin-680 conjugate (Sigma Aldrich, 2 µg/mL, 5% BSA TBST) overnight at 4 °C then washed 3X TBST then imaged on a Li-cor Odyssey CLx. Subsequently membranes were incubated with anti-NOTUM antibody (Abcam AB106448, 1:1000, 5% BSA TBST) at 4 °C overnight, washed 3X TBST and incubated with a secondary antibody (CST DyLight 800 4X PEG Conjugate anti-rabbit, 1:3000, 5% BSA TBST) for 2 hours. Transfers were then washed 3X in TBST then imaged using a Li-cor Odyssey CLx. Blots were then analysed using Image Studio Lite to calculate the optical density of the fluorescent band due to biotinylated NOTUM.

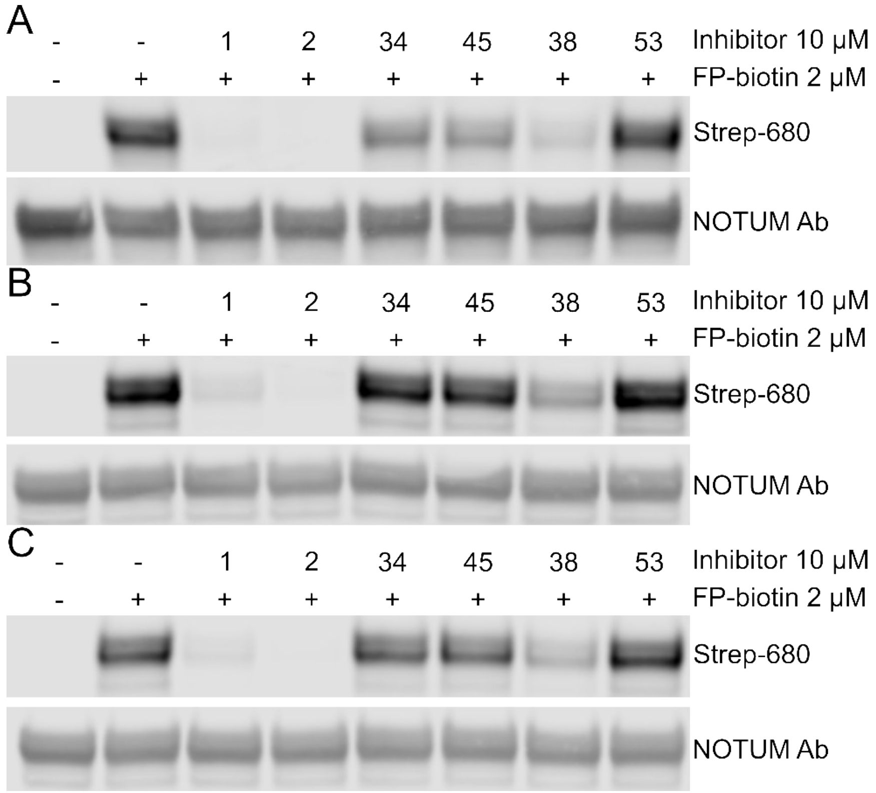

Figure S2. Blots used to generate data for FP-biotin competition assay

TCF/LEF reporter (Luciferase) cell based assay

20 μL of TCF LEF cells were plated in 384 well cell culture Greiner microplates (catalog #781098) at 10,000 cells per well and incubated overnight at 37 ^{o}C. An Echo liquid handler was used to acoustically dispense 150 nL of compounds into V-well polypropylene Greiner microplate (catalog #781280) to create a 10 point curve. 25 μL of 0.5 % DMEM media (500 mL DMEM 1X GlutaMax, 4.5 g/L D-Glucos, Pyruvate plus 2.5 mL 10 % Fetal Bovine Serum and 5 mL Penicillin Streptomycin) was added to the Wnt compound plates and 25 μL of NOTUM at 1000 μg/mL was added to the NOTUM compound plates and incubated at room temperature for 10 minutes. 25 μL of Wnt-3A at 200 ng/mL was added to all plates and incubated at room temperature for 1 hour. 20 μL of media was aspirated from the cell plates using a CyBio SELMA followed the addition of 40 μL from the compound plates. After an overnight incubation at 37 ^{o}C GFP fluorescence was measured on a PHERAstar *FSX* microplate reader with a wavelength of 458 nm and emission wavelength of 520 nm. 20 μL of steady-glo luciferase assay buffer (Promega #E2520) was added to the cell plates using the CyBio, the luminescence was measured on the PHERAstar.

Table S2. Comparison of NOTUM OPTS biochemical and TCF/LEF reporter cell based assay data for compound **1**.

Assay	This work	Literature value[Refs]
NOTUM inhibition (OPTS), IC_{50} (μM)	0.0011 ± 0.0004 (n > 10)	0.001[2]
TCF/LEF reporter (Luciferase), EC_{50} (μM)	0.138 ± 0.053 (n = 4)	-
CellSensor LEF/TCF-*bla* FreeStyle 293F cell line, EC_{50} (μM)	-	0.021[3, 4]

In vitro ADME screens

Selected compounds were screened for aqueous solubility, transit performance in MDCK-mdr1 cell lines for permeability, and metabolic stability in human and mouse liver microsomes as a measure of clearance.

ADME studies reported in this work were independently performed by GVK Biosciences (Hyderabad, India) and/or Cyprotex (Macclesfield, UK).

Cyptotex, UK. See: https://www.cyprotex.com/admepk

GVK Biosciences, India. See: https://www.gvkbio.com/discovery-services/biology-services/dmpk-services/

The MDCK-mdr1 cell line used in these experiments was supplied to GVK Biosciences from SOLVO Biotechnológiai ZRT.

Protein crystallization, data collection and structure solution:

Crystallization and Inhibitor Soaking

An optimised form of the apo-NOTUM(81-451) crystals previously reported as crystal form III (PBD ID: 4UZ1) were suitable for compound soaking and high-resolution structure determination (typically to 1.5 Å resolution)[1]. Crystallographic fragment screening at the XChem facility (Diamond Light Source beamline I04-1) was carried out essentially as described[5] using a highly streamlined process that includes acoustic droplet ejection technology to dispense fragment compounds and Diamond Light Source beamline I04-1 in unattended mode.[6] Crystals for inhibitor soaking were grown by sitting-drop vapor diffusion in 96-well plates at 22 °C, using a protein concentration of 4.5 mg/mL, 1.4 M ammonium sulphate, 0.1 M citric acid, pH 4. Compounds **39** and **45** were re-suspended in 100 % DMSO and then diluted by adding the crystallization condition buffer (supplemented with 40 % ethylene glycol) to reach a final DMSO concentration of 15 %. The crystals were then soaked for 5 min, 10 min or overnight, and subsequently flash-frozen in liquid nitrogen.

X-ray Data Collection, Data Processing, Structure Determination, and Structure Refinement

Standard methods were used for X-ray diffraction data collection and determination of the structures of compounds **39** and **45** bound to NOTUM. Briefly, datasets were collected at 100 K at Diamond Light Source with Pilatus 6M detectors at beamlines I04-1 or I24 using a wavelength of 0.91 Å or 0.97 Å, respectively. Molecular replacement using the apo-form of NOTUM (PBD ID: 4UZ1) was performed using Phaser[7], followed by iterative cycles of refinement using phenix.refine[8] and manual rebuilding using Coot[9]. Models were validated with MolProbity[10]. Compound molecules and restraints were built from SMILES using the server Grade Web Server.[11]

Table S3: Data collection and refinement statistics

	Notum_ Fragment **3**	Notum_ Benzotriazole **39**	Notum_ Isoquinoline **45**
PDB ID code	6R8P	6R8Q	6R8R
Ligand code	JVB	JV5	JV8
**Data collection**			
X-ray source (Diamond)	I04-1	I24	I04-1
Wavelength (Å)	0.92819	0.96863	0.91587
Space group	$P2_12_12_1$	$P2_12_12_1$	$P2_12_12_1$
Cell dimensions			
a, *b*, *c* (Å)	60.6, 72.8, 79.0	60.1, 71.3, 78.3	60.3, 71.7, 78.1
α, β, γ (°)	90, 90, 90	90, 90, 90	90, 90, 90
Resolution (Å)	1.45 (1.47-1.45) *	1.50 (1.52 -1.50)	1.27 (1.29-1.27)
R_{merge}	0.065 (---)	0.140 (0.941)	0.081 (---)
$I / \sigma I$	10.5 (1.1)	9.6 (1.7)	13.7 (1.1)
CC 1/2	0.999 (0.519)	0.998 (0.610)	0.999 (0.520)
Completeness (%)	100 (98.7)	99.9 (95.8)	100 (100)
Redundancy	6.3 (5.5)	12.5 (12.6)	13.0 (13.2)
**Refinement**			
Resolution (Å)	53.76-1.47	52.69 - 1.5	24.20 – 1.27
No. reflections	62462 (6100)	54513 (5288)	89931 (8890)
R_{work} / R_{free}	0.199/0.227	0.155/0.195	0.148/0.177
No. atoms			
Protein	2854	2906	2968
Ligand/other	18/90	21/74	22/114
Water	145	202	184
B-factors (Å^2)			
All atoms	24.1	21	23
Ligand	52.3	19	28
R.m.s. deviations			
Bond lengths (Å)	0.01	0.008	0.013
Bond angles (°)	1.09	1.0	1.2

*Values in parentheses are for highest-resolution shell.

References

1. Kakugawa, S. *et al.* NOTUM deacylates Wnt proteins to suppress signalling activity. *Nature* **519,** 187–192 (2015).

2. Brommage, R. *et al.* The WNT-Inactivating Lipase NOTUM as a Novel Anabolic Osteoporosis Drug Target. WCO-IOF-ESCEO World Congress on Osteoporosis, Osteoarthritis and Musculoskeletal Diseases. Malaga, Spain; April 2016. https://link.springer.com/content/pdf/10.1007%2Fs00198-016-3530-x.pdf

3. Tarver, J. E. *et al.* Stimulation of cortical bone formation with thienopyrimidine based inhibitors of NOTUM Pectinacetylesterase. *Bioorg. Med. Chem. Lett.* **26,** 1525–1528 (2016).

4. Barbosa, J. *et al.* Inhibitors of Notum pectinacetylesyerase and methods of their use. Patent Application US 20120065200.

5. Collins, P. M., Ng, J. T., Talon, R., Nekrosiute, K., Krojer, T., Douangamath, A., Brandao-Neto, J., Wright, N., Pearce, N. M. & von Delft, F. Gentle, fast and effective crystal soaking by acoustic dispensing, *Acta Crystallogr D Struct Biol.* **73**, 246-255 (2017).

6. https://www.diamond.ac.uk/Instruments/Mx/Fragment-Screening.html

7. McCoy, A. J. *et al.* Phaser crystallographic software. *Journal of Applied Crystallography* **40**, 658-674 (2007).

8. Afonine PV, Grosse-Kunstleve RW, Echols N, Headd JJ, Moriarty NW, Mustyakimov M, Terwilliger TC, Urzhumtsev A, Zwart PH, Adams PD. Towards automated crystallographic structure refinement with phenix.refine. *Acta Crystallogr D Biol Crystallogr*. **68**, 352-367 (2012).

9. Emsley, P., Lohkamp, B., Scott, W. G. & Cowtan, K. Features and development of Coot. *Acta crystallographica. Section D, Biological crystallography* **66**, 486-501 (2010).

10. Chen, V. B. *et al.* MolProbity: all-atom structure validation for macromolecular crystallography. *Acta crystallographica. Section D, Biological crystallography* **66**, 12-21 (2010).

11. http://grade.globalphasing.org/cgi-bin/grade/server.cgi
